# An Iteratively Adapted Transdiagnostic Prevention Program for Diverse High School Settings (U-PEACE): Protocol for a Randomized Controlled Trial

**DOI:** 10.2196/74080

**Published:** 2025-09-24

**Authors:** Clarissa Victoria Velez, Mileini Campez-Pardo, Jennifer Mariam Canovas, Paloma Maria Pedronzo, Yeojin Amy Ahn, Chelsea Faye Dale, Sannisha K Dale, Lisa Gwynn, Amanda Jensen-Doss, Elizabeth R Pulgaron, Sara Mijares St George, Jill Ehrenreich-May

**Affiliations:** 1 Department of Psychology University of Oregon Eugene, OR United States; 2 Department of Pediatrics Miller School of Medicine University of Miami Miami, FL United States; 3 Department of Psychology University of Miami Coral Gables, FL United States; 4 Department of Psychology Harvard University Cambridge, MA United States; 5 Department of Public Health Sciences Miller School of Medicine University of Miami Miami, FL United States

**Keywords:** adolescent, anxiety, depression, transdiagnostic, prevention, school

## Abstract

**Background:**

Despite many adolescents experiencing mental health concerns, a substantial portion lack access to evidence-based treatments (EBTs) for psychopathology; this issue is magnified for adolescents belonging to communities considered marginalized. One way to ameliorate this is by adapting existent EBTs—typically delivered in research settings—so that they are feasible and scalable in adolescent settings, such as high schools. The Unified Protocol for Transdiagnostic Treatment of Emotional Disorders in Adolescents may be particularly suited for this purpose due to its transdiagnostic, modular approach and its focus on adolescent clients.

**Objective:**

This study aimed to iteratively adapt and implement the Unified Protocol for Transdiagnostic Treatment of Emotional Disorders in Adolescents in 3 Title 1 high schools, with a focus on feasibility and scalability of the intervention in diverse high school settings.

**Methods:**

For initial adaptation, members of participating high school communities will be presented with original, unadapted intervention materials and asked to provide qualitative feedback on how to make the program more appropriate and feasible for their schools (aim 1). After initial adaptations are implemented, an open-trial pilot case series will assess the appropriateness and feasibility of the resulting program: the Unified Protocol for Emotional and Academic Challenges in Education (aim 2). Initial outcome data and qualitative feedback from pilot case series participants will then inform final adaptations for the randomized controlled trial—in which the adapted program will be compared to high schools’ mental health services as usual (aim 3). The adapted program’s effectiveness will be evaluated by using a mixed methods approach, and feasibility will be preliminarily assessed through cost-effectiveness analyses (aim 4).

**Results:**

Data collection for the study was concluded in May 2025, with primary outcome analyses anticipated to be completed by August 2025.

**Conclusions:**

This protocol may serve as a promising guide for adapting youth EBTs in more accessible, diverse settings, as well as result in a useful prevention program for youth with emotional concerns.

**Trial Registration:**

ClinicalTrials.gov NCT06056674; https://clinicaltrials.gov/study/NCT06056674

**International Registered Report Identifier (IRRID):**

DERR1-10.2196/74080

## Introduction

### Adolescent Emotional Disorders: A Public Health Concern in the United States

Adolescent emotional disorders are a serious public health concern in the United States. Rates of adolescent depression rose by 7.7% from 2009 to 2019 [1]. Adolescent anxiety has also increased in prevalence [2], and research suggests that the COVID-19 pandemic exacerbated these trends [2-4]. A report from the Centers for Disease Control and Prevention suggests that nearly all indicators of mental health struggles (eg, persistent feelings of sadness or hopelessness and seriously considered suicide) among high school students steadily increased between 2011 and 2020, and the same report demonstrated that approximately 29% of adolescents endorsed experiencing mental health struggles [5]. Notably, the pandemic has intensified stressors that disproportionately impact adolescents who hold minoritized identities—including, but not limited to, academic challenges (eg, chronic absence and widening achievement gaps in schools) [6-8], financial hardship (eg, job loss and food insecurity) [9], and experiences of racism [10]. Such phenomena underscore the need to deliver mental health care that addresses the needs of diverse adolescents.

Psychologists have created a wide array of evidence-based treatments (EBTs) for adolescent emotional disorders [11,12]. Nonetheless, the National Survey of Children’s Health [13] reports low treatment use among youth, especially those who hold minoritized identities. In 2022, only 14% of White children, 11.1% of Black children, 10.3% of Latine children, and 3.4% of Asian children received mental health treatment. Among those not receiving treatment, the percentage of caregivers who believed their child needed care but did not get it was 2.9% for White children, 3.7% for Black children, 2.8% for Latine children, and 1.6% for Asian children [13]. In the same report, 3.7% of families living at or below the federal poverty level reported not receiving mental health care despite needing it, compared to 2.1% to 2.9% of families with an income of 200% or more than the federal poverty level [13]. Barriers to use of EBTs may be practical (eg, financial barriers and transportation) [14,15] and attitudinal (eg, stigma) [16] in nature. The proximity of school environments to adolescents makes high schools a promising setting to overcome these barriers [17], especially given that adolescents’ academic performance is often interrelated with their mental health [18].

### School-Based Interventions for Youth Anxiety and Depression

Mental health professionals can address these barriers by meeting adolescents where they naturally are and delivering evidence-based mental health services in community settings—such as high schools [17,19-21]. The existing research on school-based interventions for anxiety and depression is primarily focused on increasing access to care, health promotion, and prevention [22-25]. Consequently, school-based interventions have made it possible to deliver mental health care to millions of children who likely would not have received care outside of this setting [26]. Furthermore, school-based services offer the opportunity for mental health professionals to fulfill the mental health needs of children and adolescents through training of school staff [27], increasing prevention efforts [18,24], and reduction of stigma associated with mental health issues [28].

However, while many of these interventions are found to be effective, effect sizes with school-based samples are typically smaller than those with clinic-based samples, and they are often not statistically significant in comparison to clinic-based settings [29-31]. Smaller effect sizes may, in part, be due to the challenges that mental health professionals have faced when adapting and implementing these EBTs into community settings [31-33]. Particularly, there may be school-specific barriers to implementing and sustaining some components of interventions that are more traditionally offered in clinics, such as exposures [33]. These may include financial (eg, purchase materials and pay for staff), attitudinal (eg, support of other school staff), and logistical (eg, competing school priorities) barriers that further compound misalignment between the goals of an EBT and a school’s values and focus [34]. Consequently, specific components of EBTs may not be applied with fidelity—or may be deimplemented by stakeholders altogether, given the school-specific barriers—reducing the effectiveness of the interventions [35]. Some of these implementation challenges may be mitigated by using a transdiagnostic intervention that can address a wide range of mental health concerns [36].

### Unified Protocol for Transdiagnostic Treatment of Emotional Disorders in Adolescents

Although comorbid psychiatric diagnoses are common in both clinical and school settings, and youth with these presentations often face greater impairments, many EBTs focus on disorder-specific presentations and techniques [20,37]. This problem-focused structure can result in a lower treatment response among adolescents who are most affected by their symptoms, suggesting a need for scalable school-based interventions that can address symptomatology across internalizing diagnoses [38]. Transdiagnostic interventions, such as the Unified Protocols for Transdiagnostic Treatment of Emotional Disorders [39], operate under the premise that internalizing or emotional disorders can be functionally treated as a singular syndrome, with overlapping symptoms and risk factors originating from variances in emotional reactivity and regulation [40,41]. The Unified Protocol for Transdiagnostic Treatment of Emotional Disorders in Adolescents (UP-A) [42] is an evidence-based transdiagnostic treatment that was developed concurrently with the adult Unified Protocol, and it may hold promise in addressing some of the current limitations of school-based anxiety and depression interventions. The UP-A has been found to effectively treat adolescent emotional disorders in multiple studies, including randomized controlled trials (RCTs) [43,44].

The UP-A treatment protocol has a modularized, flexible structure that allows mental health providers to address varying and comorbid emotional mental health concerns [42]. This structure reduces required training for community-based mental health providers while simultaneously increasing the number of adolescents and variety of presenting concerns that may benefit from the intervention. The UP-A has also previously been implemented in community-based mental health care settings [45,46] and in more structured, group-based formats.

### Inclusion of Community Member Input

The long-standing absence of culturally responsive treatment in diverse settings has contributed to disparities in mental health outcomes in underserved communities [47]. Furthermore, increased awareness of the limited representation of minoritized adolescents in foundational EBT research has prompted efforts to adapt existing treatments to better fit the needs and priorities of diverse adolescents [48,49]. Content adaptations of EBTs are encouraged to prioritize the alignment of diverse youth’s values, customs, goals, and traditions, with those incorporated into the intervention [50]. Similarly, cultivating community partnerships through intentional engagement of community leaders (eg, school administrators, students, and caregivers) is a key approach to gaining insights into the unique school environment; this, in turn, may aid researchers in tailoring interventions to better fit the unique needs of this setting [50]. Intervention dissemination and implementation models recommend centering the expertise of individuals who are a part of the target community in the development and implementation of interventions to overcome potential mismatch among lived experiences of diverse adolescents, treatment goals, and school goals [50-52]. Inclusion of community members’ input into the development and modification of school-based mental health interventions can help elucidate community perceptions of mental health treatment, identify and modify logistical barriers, and ensure mutual benefit of intervention implementation; this may lead to increased engagement, effectiveness, and sustainability of interventions [53,54]. Previous research has successfully incorporated community members’ feedback in the adaptation and implementation of multiple evidence-based mental health interventions for youth, including but not limited to a cognitive behavioral therapy (CBT) intervention for Haitian-American adolescents diagnosed with depression conducted in a middle school [55], digital single-session interventions for youth living in south Texas [56], and mental health treatment engagement interventions conducted in a transition resource center for youth aging out of foster care [57].

### This Study

The purpose of this study is to iteratively adapt and implement a school-based, indicated prevention version of the UP-A in Title 1 high schools that cater to minoritized youth and evaluate its efficacy within that setting. The program will be known as the Unified Protocol for Transdiagnostic Treatment of Emotional and Academic Challenges in Education (U-PEACE). First, we anticipate that verbal feedback from community members would suggest that U-PEACE is a good fit for Title 1 high schools, with the inclusion of some suggested modifications. Next, we anticipate that U-PEACE, developed via an iterative feedback process, including 3 phases of evaluation (ie, qualitative interviews of stakeholders, pilot case series, and an RCT), will lead to reductions in anxiety and depression symptoms, as well as improvements in academic domains (eg, grades and attendance). Finally, we anticipate that adoption of U-PEACE will be more cost-effective for high schools than services as usual (SAU) within integrated school health clinics.

## Methods

### Study Design

#### Setting and Community Partnership

The University of Miami School Health Initiative clinics provide comprehensive health and mental health services in the 3 Title 1 public high schools where the research protocol will be implemented. These school clinics offer integrated mental health treatment and consultation alongside the provision of no-cost primary and preventive medical care to students attending the schools. The school health clinics have provided these services for >20 years through local grants in collaboration with the high schools. Per the most recent statistics from the National Center for Education Statistics [58], 4249 students attend these schools, of which 66% are Black and 31% are Hispanic. Approximately 66% of students within these schools qualified for free or reduced lunch, indicating high financial need during the 2019 to 2020 school year [58]. Rather than establishing an independent community partnership with local Title 1 high schools, the research team will leverage the existing, long-standing partnership between these clinics and the 3 high schools to carry out this project.

#### Participants

Participants will be students enrolled at the 3 participating underserved (ie, Title 1) high schools. School staff members (eg school counselors and teachers) and caregivers will also be asked to participate by providing feedback on the mental health and program engagement of the students enrolled in U-PEACE. We anticipate the students enrolled in our study to be demographically representative of the high schools they attend.

#### Recruitment

High school student participants will be recruited through staff referrals and community outreach. Students will also be able to express interest through QR codes on flyers posted throughout the high school campuses and on high school social media pages. Students can qualify for U-PEACE if they are aged between 13 and 18 years at the time of enrollment into the program and if they self-report clinically significant depressive symptoms (as indicated by scores at or above the clinical cutoff for the Patient Health Questionnaire-9, PHQ-9 [59,60]) or clinically significant anxiety symptoms (as indicated by scores at or above the clinical cutoff for the Generalized Anxiety Disorder-7 [61,62]). To qualify for U-PEACE, students will also need to be enrolled at one of the target high schools, want to voluntarily participate, and have a caregiver who will be available and willing to voluntarily sign their consent forms.

In addition to students who do not meet inclusion criteria, students will be excluded if their psychological symptomology is considered too severe to be effectively treated with a school-based prevention program, if they have neurological or medical conditions that may impact their ability to effectively participate in the group program, or if they do not speak or understand English or Spanish. During phases 2 and 3 of the study, students will also be excluded if they have a self-reported history of seizures, autism spectrum disorder, substance use disorder, serious mental illness, or other cognitive delay.

Staff members will be recruited to participate as support staff (eg, coach) through recommendations by individuals that participated in the qualitative interviews, clinic staff, or the administrators at participating schools. Staff at the target high schools will be excluded if they are not aged at least 18 years or are not able to speak and understand English fluently.

### Ethical Considerations

This study, with the stated methods, is approved by the University of Miami institutional review board (20220700, 20230796). Recruitment and implementation efforts within the participating schools were approved by the school district’s Research Review Committee. Informed written consent will be obtained from the caregiver or caregivers of minor adolescent participants and directly from the adolescent participants themselves if they are aged 18 years (for sample consent forms, refer to Multimedia Appendices 1 and 2). Furthermore, informed written assent will be obtained from adolescent participants aged <18 years. To ensure understanding of consent materials, these materials will be provided in English or Spanish and bilingual staff will be available to respond to questions about the study. Students will receive US $50 at each time point for completion of questionnaires and assessments, except at the midpoint when they will receive US $25. Teachers (ie, school staff who complete the Adolescent Academic Problems Checklist [AAPC]) will receive US $5 for each questionnaire completed at each time point. Caregivers will receive US $10 for questionnaires completed at each time point. Coaches will receive US $20 for each student for whom they complete more than 50% of their questionnaires between baseline and midpoint and another US $20 for each student for whom which they complete more than 50% of their questionnaires between midpoint and after treatment.

### Study Timeline and Aims

**Figure 1:**
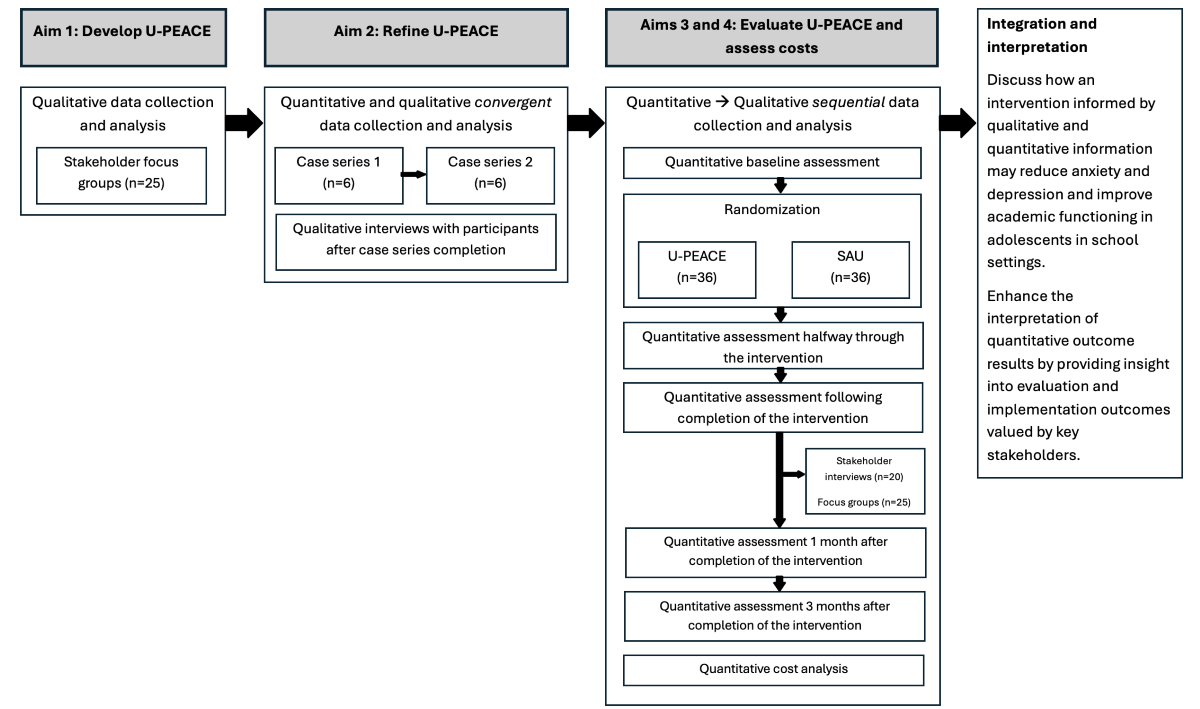
Unified Protocol for Transdiagnostic Treatment of Emotional and Academic Challenges in Education (U-PEACE) study design and evaluation flowchart. SAU: service as usual.

This study has 3 distinct phases to accomplish 4 primary aims using a hybrid-type 1 effectiveness-implementation design [63]. In phase 1, we will interview community members for feedback on (1) mental health needs of adolescents at the target schools and (2) suggestions for delivering transdiagnostic therapeutic services in a high school setting (aim 1). The feedback obtained will be used to inform adaptations for the first iteration (pilot case series) of U-PEACE (school-based UP-A). In phase 2, we will initiate an open-trial pilot case series study with the first iteration of U-PEACE. The pilot case series will be initiated to assess the feasibility of program implementation and identify additional implementation barriers to be remedied in the second iteration (RCT). Feedback from interviews with participants in the pilot case series and support personnel for U-PEACE implementation will be incorporated into U-PEACE’s content and delivery (aim 2). In phase 3, we will initiate a randomized, controlled effectiveness trial (RCT) in which the second iteration of U-PEACE will be compared to mental health services as usual in Title 1 high schools (aim 3). In addition, in phase 3, we will analyze U-PEACE implementation (eg, adherence to U-PEACE and session attendance), collect additional feedback through qualitative interviews with study participants who received U-PEACE, and analyze cost-effectiveness (aim 4). For a flowchart of the study timeline and aims, refer to Figure 1.

### Feedback and Source of Adaptations

#### The Unified Protocol for Transdiagnostic Treatment of Emotional Disorders in Adolescents

The original UP-A treatment guide follows a CBT framework. However, rather than targeting disorder-specific symptomatology as is the case in traditional CBT protocols, it focuses on higher-order factors that underlie a range of emotional disorders, such as emotion regulation and distress tolerance [42]. The UP-A follows a flexible, modularized structure; when in group format in research clinics, its 8 modules are typically delivered across sixteen 90-minute sessions. For a review of each of the modules in the UP-A, refer to Table 1 [42].

When adolescents are enrolled in the original UP-A treatment, they receive a workbook that consists of both session worksheets (designed to facilitate content learning) and homework worksheets. Adolescents are expected to complete homework practice between each UP-A session to help them apply the skills learned in UP-A outside of the group. While caregivers do not have a regularly scheduled UP-A caregiver group, they are traditionally expected to be engaged and participate within the program. For example, caregivers are provided with session materials and may be given a brief overview by clinicians of content covered within the session. Caregivers are also asked to help their adolescent complete homework between sessions.

**Table 1:** Unified Protocol for Transdiagnostic Treatment of Emotional Disorders in Adolescents overview.

Module	Title	Recommended sessions, n	Module content
1	Building and keeping motivation	1 or 2	Build rapport with your adolescent client Discuss key problems and set goals Determine what motivates the adolescent to change
2	Getting to know your emotions and behaviors	2 or 3	Provide psychoeducation about different emotions Discuss the purpose of emotions Introduce the 3 parts of an emotion Introduce the cycle of avoidance and other emotional behaviors
3	Introduction to emotion-focused behavioral experiments	1 or 2	Introduce the concepts of opposite action and emotion-focused behavioral experiments Teach the adolescent how to track emotion and activity levels Engage the adolescent in emotion-focused behavioral experiments for sadness (and potentially other emotions)
4	Awareness of physical sensations	1 or 2	Review the connection between physical feelings and strong emotions Develop the adolescent’s awareness of their physical feelings Conduct sensational exposure exercises to help the adolescent learn to tolerate uncomfortable physical feelings
5	Being flexible in your thinking	2 or 3	Develop the adolescent’s ability to think flexibly about emotional situations. Introduce common “thinking traps” (ie, cognitive distortions) Link thoughts to actions by teaching Detective thinking and problem solving skills
6	Awareness of emotional experiences	1 or 2	Introduce and practice present-moment awareness Introduce and practice nonjudgmental awareness Conduct generalized emotion exposures by asking the adolescent to practice awareness skills when exposed to general emotional triggers
7	Situational emotion exposure	>2	Review skills the adolescent has learned in treatment so far Discuss the rationale for situational emotion exposures, introduced to the adolescent as another type of behavioral experiment Conduct situational emotion exposures in session and assign additional exposures for home learning
8	Reviewing accomplishments and looking ahead	1	Review skills and progress toward goals Create a relapse prevention plan
P	Parenting the emotional adolescent	1-3	Build parent awareness of responding to the adolescent’s distress Introduce 4 common emotional parenting behaviors and their opposite actions (opposite parenting behaviors)

#### Initial Development of the Unified Protocol for Emotional and Academic Challenges in Education

Program content will be adapted by a team led by the last author, who was the creator of the original UP-A. Adaptations will be focused on using the protocol as an indicated prevention program, improving the cultural acceptability of content, and increasing logistical feasibility of delivery within a dynamic, Title 1 high school setting. Initial adaptations of the UP-A will be informed by semistructured qualitative interviews of members of the school communities conducted within phase 1. These members will include caregivers, students, teachers, administrators, and mental health providers at the schools. We will aim to recruit 5 of each type of school community member, with representatives from each of the high schools participating in the study. The interviews will be conducted as focus groups or individual interviews held online via Zoom (Zoom Communications, Inc) or in person at the schools.

During the interviews, the research team will provide interviewees with examples of the UP-A and ask for feedback on how to make the program better suited for Title 1 high school settings that serve minoritized populations. Interviewees will be asked questions regarding content fit (eg, How can we make U-PEACE more relevant to teens in your school?) and feasibility of delivery (eg, When should we deliver U-PEACE in schools?). The interviews will incorporate an overview of the UP-A, a sample outline of an UP-A session, and a demonstration video for the interviewees’ reference. The treatment development team will use the community member feedback to adapt the content and delivery of U-PEACE for implementation into the Title 1 high school communities [64].

#### Iterative Feedback

Throughout phases 2 and 3 of the study, feedback will be iteratively requested from community members through semistructured qualitative interviews and advisory committee meetings.

In addition to obtaining feedback about initial adaptations to the UP-A, feedback will also be requested at the end of phase 2 by individuals who participated in the study. These interviewees may consist of caregivers, students, and mental health care providers. Participants in the U-PEACE pilot case series will be asked to reflect on their experiences in the program and provide feedback on (1) how the program impacted them, (2) how fitting the program was for them and members of their community, and (3) whether the logistics of the program were feasible. On the basis of phase 2 feedback, the research team will adapt the content and delivery of U-PEACE for implementation during the RCT phase of the study. This second iteration of adaptations will again be focused on improving the cultural acceptability of content and increasing logistical feasibility of delivery at the participating Title 1 high schools.

Some participants and their caregivers who are randomized to the U-PEACE group during the RCT phase (phase 3) of the study will be randomly selected to participate in semistructured qualitative interviews at the end of their participation in the program (for a sample interview guide, refer to Multimedia Appendix 3). Participants will consist of an even distribution of responders and nonresponders to the U-PEACE program. Interview questions will mirror questions asked of the pilot case series participants. The third iteration of adaptations will have the same focus as the previous adaptations, with an added focus of making the program scalable and deliverable in similar Title 1 high school settings. The RCT feedback (following completion of phase 3) may also provide guidance on how to further scale the implementation and delivery of U-PEACE in other high school settings.

In addition to qualitative interviews, the research team will also ask the Title 1 high school communities to identify members who are well-known on their campuses and engaged in the community to participate in an advisory committee. Members will ideally include teachers, students, caregivers, school mental health providers, and administrators from each of the high schools. Members will meet at the end of each of the first 3 study aims. During the advisory committee meetings, members will be updated on the study’s status and asked to provide feedback. Community members that participate in qualitative feedback interviews or advisory committee meetings will receive US $50 compensation for each time they participate in providing feedback.

### Prevention Program Content and Delivery

#### Content

The resulting U-PEACE indicated prevention program is anticipated to maintain the same overarching intervention principles and flexible modules as the UP-A. Content within existing UP-A modules may be adapted (eg, examples of skills may be more tailored to reflect common adolescent experiences in Title 1 high schools), and modules may be added or removed based on community member feedback. Like the UP-A, each module will have corresponding session materials that facilitate content learning and encourage practice of skills outside of the U-PEACE group. In addition to clinician, adolescent, and caregiver materials; we also anticipate materials to be created for school community members (eg, teachers, school mental health providers, or counselors) who may be involved in facilitating adolescents’ practice of skills or homework completion.

#### Study Intervention

**Figure 2:**
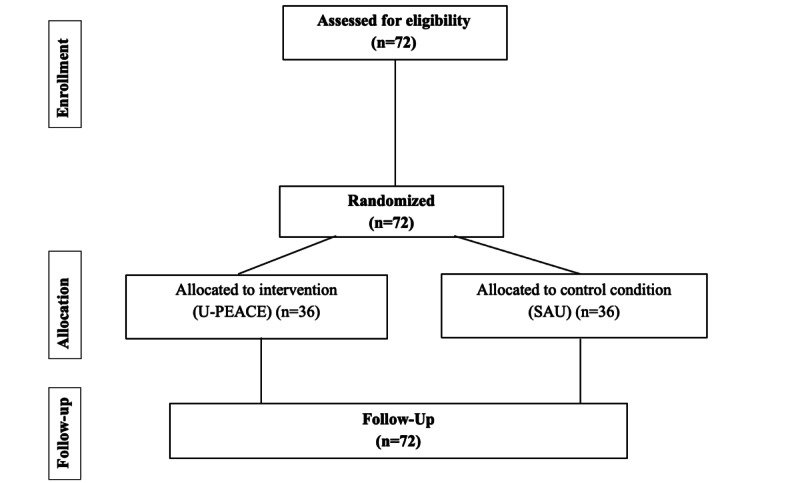
Anticipated CONSORT (Consolidated Standards of Reporting Trials) diagram for the Unified Protocol for Transdiagnostic Treatment of Emotional and Academic Challenges in Education (U-PEACE) study. The diagram includes information from the anticipated participant allocation. At the completion of the study, a final CONSORT diagram will be presented with study results that include final data regarding recruitment and retention of participants. SAU: service as usual.

Given that the study was already in phase 3 when this manuscript was submitted for publication, the written protocol for U-PEACE delivery will incorporate community member feedback from phases 1 and 2. For a CONSORT diagram of the RCT, refer to Figure 2.

While the research team will be exploring the feasibility of telehealth-delivered U-PEACE, most U-PEACE sessions are expected to be delivered in person, during lunchtime, and in group format. Students who are enrolled in U-PEACE will be permitted to access additional primary, medical, and mental health care services within the school clinics throughout their participation in U-PEACE. The original UP-A typically takes place over 12 to 16 sessions. Thus, it was anticipated that U-PEACE would eventually contain a session count in this range. Each U-PEACE group will ideally have approximately 4 to 8 students enrolled in it at one time, as well as 1 to 2 clinicians in training who serve as program leaders for the group. At the beginning of the U-PEACE group, adolescent participants will receive a binder of U-PEACE session and homework worksheets that they can reference when learning and reviewing the program content. Adolescents will be expected to bring their binders to every U-PEACE session and practice skills learned in the group as homework between program sessions. In addition to U-PEACE binders, adolescents will also be given a caregiver session summary sheet to provide to their caregivers; these summary sheets are intended to keep caregivers informed about skills their adolescents are learning in U-PEACE.

Although initial community member feedback suggested that the research team bring external clinicians into the schools to deliver U-PEACE, the team decided to also incorporate nonclinic school staff into the delivery of U-PEACE by adding coaches to the program. Coaches were typically existent school counselors or school mental health providers at the target schools who are intended to function in U-PEACE in a similar manner to how caregivers function in the UP-A: their primary role is to review and practice skills learned in U-PEACE sessions with adolescents outside of the program (eg, ensuring homework completion). Coaches will try to meet with each assigned student one time per week for approximately 15 minutes. During the meeting, coaches will briefly check in with students regarding whether they have been able to practice the skills they learned in session. They will also provide students with session summary materials if they cannot attend the previous session.

#### Comparison Condition

Individuals assigned to the comparison condition will have access to any medical, social work, and psychotherapy services available through the school clinic to address emotional and behavioral concerns determined to be needed during the intervention period. The school clinic services are available in person or via telehealth throughout the year. A study staff member will also meet with all SAU adolescents for approximately 10 minutes per week (via telehealth as needed) for 13 weeks to complete weekly study measures, assess and provide linkage to clinic services requested or needed, and monitor for clinical deterioration. If an adolescent seems to be deteriorating in any significant way, additional mental health services or appropriate referrals will be offered after consultation. Data will also be collected from SAU participants 1-month after completion of the 13 weekly meetings. After the 1-month follow-up, SAU participants may be offered participation in the U-PEACE program via telehealth or in person.

SAUs vary in frequency and type based on the availability of the school clinic providers. These services may include weekly individual or group CBT, case management, and psychological and psychiatric consultations. These services are often provided by nurses, primary care physicians, master’s-level therapists, social workers, psychologists, or psychiatrists. The receipt of these services within the school clinic will be monitored and reported. If a higher level of care is needed than what is typically provided through the school clinic, patients are referred to community resources. Participants will also be asked to report on their access and use of emotional and behavioral health services outside of the school clinic.

### Measures

#### Overview

Each U-PEACE and SAU group will have 4 assessment time points (baseline, midpoint, after completion, and 1-month follow-up) of questionnaire administration. Detailed information regarding questionnaire administration at each time point can be found in Table 2.

**Table 2:** List of assessment measures and time points.

Measure name	Every session	Baseline	Midpoint	After completion	1-month follow-up
Demographic survey	N/A^a^	Adolescent and parent or caregiver	N/A	N/A	N/A
DIAMOND-KID^b^–full interview [65]	N/A	Adolescent	N/A	N/A	N/A
DIAMOND-KID–initial interview [65]	N/A	Adolescent^c^	N/A	N/A	N/A
DIAMOND-KID–self-report scanner [65]	N/A	Adolescent^d^	Adolescent^d^	Adolescent^d^	Adolescent^d^
Service Utilization Form	N/A	Adolescent^d^	Adolescent^d^	Adolescent^d^	Adolescent^d^
Generalized Anxiety Disorder 7-item Scale [61]	N/A	Adolescent and parent or caregiver	Adolescent and parent or caregiver	Adolescent and parent or caregiver	Adolescent and parent or caregiver
Child Anxiety Interference Scale–Academic Subscale [66,67]	N/A	Adolescent and parent or caregiver	Adolescent and parent or caregiver	Adolescent and parent or caregiver	Adolescent and parent or caregiver
Patient Health Questionnaire-9 [59]	N/A	Adolescent and parent or caregiver	Adolescent and parent or caregiver	Adolescent and parent or caregiver	Adolescent^e^ and parent or caregiver
Affective Reactivity Index [68]	N/A	Adolescent and parent or caregiver	Adolescent and parent or caregiver	Adolescent and parent or caregiver	Adolescent and parent or caregiver
Adolescent Academic Problems Checklist [69]	N/A	Teacher	Teacher	Teacher	Teacher
The Revised Children’s Anxiety and Depression Scale–Short-Form [70]	N/A	Adolescent^d^	Adolescent^d^	Adolescent^d^	Adolescent^d^
Behavior and Feelings Survey [71]	N/A	Adolescent^d^	Adolescent^d^	Adolescent^d^	Adolescent^d^
School achievement (as measured by attendance and report card)	N/A	Adolescent^d^	N/A	Adolescent^d^	Adolescent^d^
Everyday Discrimination Scale [72]	N/A	Adolescent	Adolescent	Adolescent	Adolescent
Abbreviated Multidimensional Acculturation Scale [73]	N/A	Adolescent	Adolescent^c^	Adolescent^c^	Adolescent^c^
Distress Tolerance Scale [74]	N/A	Adolescent	Adolescent	Adolescent	Adolescent
Top problems [75]	Adolescent	Adolescent	Adolescent	Adolescent	Adolescent
Participant adherence	Clinician	N/A	N/A	N/A	N/A
Therapeutic Alliance Scale for Children–Revised [76,77]	N/A	N/A	Adolescent	N/A	N/A
Client Satisfaction Questionnaire [78]	N/A	N/A	Adolescent	N/A	N/A

^a^N/A: not applicable.

^b^DIAMOND-KID: Diagnostic Interview for Anxiety, Mood, and OCD-Related Neuropsychiatric Disorders: Child and Adolescent Version—Full Interview.

^c^Not administered at these time points in the randomized controlled trial.

^d^Measure was only administered during the randomized controlled trial.

^e^Patient Health Questionnaire-8 was used at 1 month.

#### Clinical Presentation and Program Fit

To determine whether students will be a good fit for the U-PEACE program, members of the research team will conduct a modified version of the Diagnostic Interview for Anxiety, Mood, and OCD-Related Neuropsychiatric Disorders: Child and Adolescent Version (DIAMOND-KID) [65] with preliminarily eligible adolescents at baseline. The DIAMOND-KID is a semistructured diagnostic interview and self-report screener assessing clinically significant mental health concerns from the *Diagnostic and Statistical Manual of Mental Disorders, Fifth Edition* [65]. The DIAMOND-KID has demonstrated very good to excellent test-retest reliability and good convergent validity with youth aged between 10 and 17 years [65]. Inter-rater reliability on most diagnoses was found to be good to very good—except for anxiety and depressive disorders, in which inter-rater reliability was found to be questionable in youth, possibly due to inconsistent reporting of the specific duration and timing of symptoms by youth [65]. All DIAMOND-KID assessments will take place in person, on school campuses; and each assessment is expected to take about 1 hour.

#### Demographics and Service Use

Self-reported demographic information of students, caregivers, and coaches will be collected. Demographic characteristics will include contact information, age, race and ethnicity, self-reported gender, and sexual orientation. In addition, students will be asked to report on any past or current psychiatric or psychological services, as well as any past or current psychiatric medications. At each time point following baseline, students will be asked to update previously reported service and medication information. It is anticipated that both English and Spanish speaking students will be included in the RCT.

#### Academic Progress

Student grade point average for each academic quarter will be calculated by converting all grades to a 5-point scale (4.0=A to 0.0=F). Student grades will not be weighted. School attendance will be collected as measured by the number of days a student was present in school for the academic year. Both student grade point averages and attendance records will be collected in person by having students log into their web-based academic portals and share the pertinent information with study coordinators.

Academic problems will also be measured using the AAPC [69]. The AAPC is a 24-item teacher-report measure that assesses adolescents’ concerns in the classroom (eg, behavioral withdrawal and class disruptions). Items are rated on a 4-point Likert-type scale that ranges from “not at all” to “very much,” with higher scores suggesting more severe classroom concerns. The AAPC has demonstrated excellent internal consistency (0.92) and strong concurrent validity in assessing behaviors associated with academic functioning [69]. The AAPC will be distributed to teachers chosen by their respective students, with the same teacher completing the form for the same student throughout the study.

#### Anxiety and Depression Symptoms

Adolescents’ anxiety symptoms will be measured using the Generalized Anxiety Disorder 7-item Scale (GAD-7) [61]. The GAD-7 will be used as both a self- and caregiver-report measure; the items in it are rated on a 4-point Likert-type scale that ranges from “not at all” to “nearly every day,” with higher scores suggesting more severe anxiety symptoms. While the authors are not aware of published psychometric properties on the parent-report GAD-7, the self-report GAD-7 has demonstrated good reliability in adolescent populations (Cronbach α=0.88) and has demonstrated strict measurement invariance by adolescents’ sex and grade, as well as good convergent and discriminant validity [62,79].

Adolescents’ depression symptoms will be measured using the PHQ-9 [59]. Items on the PHQ-9 scale are rated on a 4-point Likert-type scale that ranges from “not at all” to “nearly every day,” with higher scores suggesting more severe depression symptoms. The PHQ-9 has demonstrated good internal reliability (McDonald Ω=0.87) among adolescents, and confirmatory factor analysis for a 1D model suggested adequate fit [80].

There may be instances in which the research team chooses to administer a modified version of the PHQ-9 to adolescents and their caregivers. For instance, given that this measure may be administered remotely without the presence of a study team member, the Patient Health Questionnaire-8 (PHQ-8) may be used to ensure that any suicide-related concerns are handled immediately and in person by the research team [60]. Research has suggested that omitting the suicide item on the PHQ-9 has minimal impact on scoring [81]. The correlation between the PHQ-9 and PHQ-8 remains high; therefore, the cutoff scores remain unchanged [60,81]. While the authors are not aware of published psychometrics for parent-report PHQ-8, research on the general population suggests good sensitivity and specificity [60].

In addition to the GAD-7 and PHQ-9, adolescents’ anxiety and depression symptoms will also be measured by the Revised Children’s Anxiety and Depression Scale–Short-Form (RCADS-SF) [70]. The RCADS-SF is only used as a self-report measure in this study. The 25 items in it are rated on a 4-point Likert-type scale that ranges from “never” to “always,” with higher scores suggesting more severe symptoms of anxiety and/or depression. The RCADS-SF has demonstrated adequate reliability and acceptable discriminant validity in comparison to the 47-item RCADS [70].

#### Anxiety-Based Impact on Academic Functioning

The impact of adolescents’ anxiety symptoms on their academic functioning will be measured by the Child Anxiety Impact Scale–Academic Subscale (CAIS-AS) [66,67]. The CAIS-AS will be used as a self- and caregiver-report measure. The 10 items in the subscale are rated on a 4-point Likert-type scale that ranges from “not at all” to “very much,” with higher scores suggesting more anxiety-based academic impairment. The CAIS-AS child report has demonstrated excellent internal consistency (0.82) and good construct validity. Similarly, the parent report exhibited very good internal consistency (0.85) and good construct validity [67].

#### Irritability

Adolescents’ irritability will be measured using the Affective Reactivity Index (ARI) [68]. The ARI will be used as a self- and caregiver-report measure. The 7 items in it are rated on a 3-point Likert-type scale that ranges from “not true” to “certainly true,” with higher scores suggesting more irritability. The ARI has demonstrated good to excellent internal consistency in both the caregiver-report and the youth self-report versions [68].

#### Internalizing and Externalizing Symptoms

Adolescents’ internalizing and externalizing symptoms will be measured using the Behavior and Feelings Survey (BFS) [71]. The BFS is only used as a self-report measure in this study [71]; it is a brief 12-item scale that ranges from “not a problem” to “very big problem,” with higher scores suggesting more severe internalizing and/or externalizing symptoms. The BFS has demonstrated good internal consistency (0.87) and strong discriminant validity [71].

#### Distress Tolerance

Adolescents’ emotional distress tolerance will be measured using the Distress Tolerance Scale (DTS) [74]. The DTS is only used as a self-report measure in this study. The 15 items in it are rated on a 5-point Likert-type scale that ranges from “strongly disagree” to “strongly agree,” with higher scores suggesting lower distress tolerance. The DTS has generally demonstrated adequate to good internal consistency (0.72-0.82) [74], and its factor structure was upheld in a validation study with children and adolescents [82].

#### Acculturation

Adolescents’ acculturative experiences will be documented using the Abbreviated Multidimensional Acculturation Scale (AMAS-ZABB) [73]. The AMAS-ZABB is a 42-item self-report measure that assesses cultural identity, language competence, and cultural competence related to acculturation in the United States and individuals’ country of origin (as applicable). Items are rated on a 4-point Likert-type scale that ranges from “strongly disagree” to “strongly agree.” While the AMAS-ZABB has been validated in some adult populations, the scale is yet to be validated in adolescents [83]. The AMAS-ZABB will be distributed to all participating students at the 4 major time points during phase 2 and only administered at baseline during phase 3 of the study.

#### Discrimination

Adolescents’ experiences of discrimination will be measured using the Everyday Discrimination Scale (EDS) [72]. In addition to assessing discrimination experiences, the EDS also assesses unfair treatment and perceived reasons for such treatment (eg, race and sexual orientation). The EDS is a 10-item self-report measure and is rated on 6-point Likert-type scale that ranges from “never” to “almost every day,” with higher scores suggesting more frequent experiences of discrimination. Although the EDS was not originally created for adolescents, the scale has been used previously in studies with adolescent participants [84].

#### Treatment Progress

Adolescents will identify 3 problems they want to target during the study using the Top Problems Assessment [75]. The Top Problems Assessment is a personalized change measure used extensively with the UP-A to assess change over time in client-derived reasons for treatment. The Top Problems Assessment has demonstrated criterion validity, test-retest reliability, and sensitivity to change with youth aged between 7 and 13 years [75]. Although the authors are unaware of a validation study of Top Problems with adolescents, Top Problems has been used with adolescent clients receiving the UP-A in prior research [85]. In this study, adolescents will provide a score of 0 to 10, with 10 being the most severe, at all 4 major time points and each program session.

#### Adherence and Fidelity

U-PEACE adherence will be measured through adolescents’ attendance in the program. Session attendance will be recorded through a single item on a clinician-reported or research team member-reported measure. Attendance at individual meetings with the program coach will also be recorded through a single item on coach-reported documentation. Homework completion will be measured through a single item on a clinician-reported measure for each treatment session. Treatment progress, attendance, and homework completion measures will only be completed by students who are randomized to the U-PEACE group. Clinician fidelity to the U-PEACE protocol will be evaluated through audio recordings or live coding by a research associate for assessment of program fidelity, upon obtaining consent from participants.

#### Therapeutic Alliance and Program Satisfaction

Adolescents’ rapport with their program leaders will be measured using the Therapeutic Alliance Scale for Children–Revised (TASC-R) [76,77]. The TASC-R is a 12-item self-report measure of therapeutic alliance. Items are rated on a 4-point Likert-type scale that ranges from “not at all” to “very much,” with higher scores suggesting a stronger therapeutic alliance. The TASC-R has been used to assess alliance in various studies, including CBT for anxiety in youth [86].

Adolescents’ satisfaction with the U-PEACE program will be measured using the Client Satisfaction Questionnaire (CSQ) [78]. The CSQ is an 8-item self-report measure of client program satisfaction, client self-efficacy, and likelihood of recommending the program to others. Items are rated on a 4-point Likert-type scale, with higher scores suggesting higher client satisfaction. The CSQ demonstrated excellent internal consistency and good convergent validity with other similar measures administered to adolescents [87,88]. Therapeutic alliance and program satisfaction measures will only be completed by students who are randomized to the U-PEACE group.

### Data Analytic Plan

#### Qualitative Analyses

Interviews will be transcribed verbatim, anonymized, and analyzed thematically using rapid qualitative analysis (RQA) [89,90]. RQA is the recommended method to conduct efficient qualitative analysis to address rapidly changing issues, such as modifying interventions to be culturally and contextually relevant in a timely manner [89,91]. The goal of RQA is to rigorously identify actionable data rather than in-depth and theoretical understanding [90]. Individual members of the investigative team summarize key findings and then audit the summaries to assess accuracy and display responses in a matrix. Responses are then discussed in team meetings to identify overlapping and divergent themes. A thorough description of the qualitative methods and findings from the initial qualitative interviews has been published previously by our team [64].

#### Quantitative Analyses

Quantitative analyses will be conducted to assess (1) within-participant treatment-related changes in emotion regulation and symptoms after participation in U-PEACE and (2) implementation outcomes, including treatment fidelity, acceptability, and implementation costs. We will examine missing data patterns, attrition rates, and distributional properties of measures and use transformations when necessary. Depending on patterns of missing data, analyses may use full maximum likelihood estimation, multiple imputation, and inclusion of covariates related to missingness to account for missing data.

Reliable change indices (RCIs) will be calculated by dividing the within-respondent differences in self-report and caregiver scores (ie, anxiety symptoms, depression symptoms, irritability, anxiety-related impairment, and academic problems) from baseline to after treatment by the SE for each measure [92]. RCI has previously yielded reliable estimates of clinically significant change over the course of treatment and can successfully analyze small sample sizes [93]. RCI >1.96 indicates clinically significant change throughout the course of the program—which would provide preliminary support that U-PEACE is impacting theoretical mechanisms and participants’ outcomes.

Furthermore, multilevel models will be evaluated to consider nesting of repeated measures (3 or 4 time points, depending on the measure) within participants, who are further nested within study conditions. School and U-PEACE group provider or providers will be included as predictors of intercept and slope to control for environmental differences between the groups. The best-fitting model for change over time will be determined for each dependent variable based on the Akaike Information Criterion and Bayesian Information Criterion. Time will be coded in weeks and will be “centered” alternately at treatment follow-up, such that model intercepts will represent mean scores at these assessments [94]. Treatment condition (ie, U-PEACE or SAU) will be entered as a predictor of the level 2 growth curve parameters (intercepts and slopes). These models will be used to assess group differences in both purported mechanisms of change (ie, emotion regulation measures) and in program outcomes (ie, symptom measures, academic outcomes, and diagnostic measures) as evidenced by slope or mean level differences at different time points.

Growth curve parameters will be used to categorize individual participants’ outcomes (as deteriorated, unchanged, improved, or recovered) by comparing them to each outcome measure’s clinical cutoffs and RCIs. Categorizations will be used to communicate findings to stakeholders and determine an appropriate range of participants for interviews after the RCT.

Implementation outcomes will be assessed using descriptive data, such as frequency of clinician adherence to U-PEACE components, participant’s homework completion, and participant’s session attendance. In addition, multilevel models (like those previously described) will be used to compare participants’ therapeutic alliance (TASC-R), program satisfaction (CSQ), and use of medication and supplemental services across study conditions.

#### Mixed Methods Analyses

Qualitative feedback, individual participant response to treatment, and treatment implementation measures from the case series will be interpreted together to elucidate the association between how experiences and perceptions of U-PEACE vary by treatment outcome and implementation. This information will be used to modify U-PEACE before the implementation of the RCT. RQA [89,90,95] will be used to quickly and efficiently summarize qualitative feedback from participants and community members throughout the trial. A joint display analysis will be developed following best practices [96]. The joint display will simultaneously illustrate individual quantitative (eg, emotion regulation, diagnostic symptomatology such as depression and anxiety, and culturally relevant outcomes such as experiences of discrimination) and qualitative (eg, themes and quotes) results reflecting participants’ experiences of U-PEACE. Likewise, we will simultaneously examine quantitative (eg, fidelity) and qualitative (eg, themes) implementation data to guide protocol changes before the RCT; these changes will be aimed to increase U-PEACE feasibility and appropriateness.

For the RCT, patterns in the quantitative outcome and implementation data will be examined to generate specific questions for the qualitative interviews after the RCT. Multilevel modeling will be used to elucidate treatment response classifications and will be used to ensure an appropriate representation of adolescent and caregiver participation in the interviews. The resulting joint display will compare representative interview quotes across key domains (eg, intervention acceptability and tolerability) and implementation indicators (eg, U-PEACE fidelity and alliance) between those classified into 2 aggregated clusters: nonresponders, which consists of those identified as falling within the deteriorated or unchanged categories, versus responders, which will include participants whose scores fall within the improved or recovered categories. Inclusion of implementation outcomes (ie, fidelity to treatment, treatment adherence, and acceptability) will provide insight into the sustainability of U-PEACE in diverse high school settings.

#### Sample Size and Power Analysis

The recommended size for a single focus group is 6 to 8 participants for sufficient diversity of opinions and to encourage discussion [97,98]. Consequently, we will seek to have at least 5 participants included in each adolescent, caregiver, teacher, school administrator, and school mental health provider focus group, for 25 participants at the initial adaptation phase, to gather sufficient data to inform the modifications to the UP-A (aim 1), rather than powering for specific effects. In addition, qualitative feedback will be obtained following the pilot case series and RCT (aims 2 and 3) to inform implementation outcomes (aim 4). All participants in the pilot case series will be invited to participant in the qualitative feedback interviews, and the same recommendation size of 25 participants will be used for the RCT qualitative feedback interviews. Power calculations for the nested multilevel models were conducted using Optimal Design 3.1 [99] to identify the minimum number of participants needed to participate in the RCT across 7 treatment groups with 5 assessment points. We estimated power using intraclass correlation coefficient (ICC) values of 0.01 based on recent effectiveness trials with a similar transdiagnostic intervention [100] and a more conservative estimate of 0.10. With an estimated sample size of 63 adolescents, the sample would have sufficient power (0.80) to detect an effect size of *d*=0.94 (ICC=0.01) or 1.21 (ICC=0.10), which are smaller effect sizes (*d*=1.25) than those observed in an UP-A RCT [43].

### Cost Analysis

While the results are expected to be preliminary and descriptive, estimates of the intervention’s total cost will be calculated. The total cost of personnel will be calculated by multiplying each personnel’s time spent on intervention activities (eg, intervention session delivery, supervision, maintenance of school engagement, and personnel travel) by their respective wages, then summing the resulting products. Administrative records of the project’s financial spending will be used to calculate nonpersonnel costs. Total costs for intervention implementation into each school will be calculated, in addition to the average cost of providing each adolescent with services. Implementation costs (cost for starting U-PEACE in schools, such as costs to initially train clinicians) will be analyzed separately from sustainability costs (cost for continuing to deliver U-PEACE in schools, such as the cost of previously trained clinicians continuing to deliver U-PEACE).

## Results

This study was funded from July 2022 to July 2025 and is being conducted over the course of 3 years. Participant recruitment and data collection for aim 1 began in September 2022, and qualitative results from aim 1 were published in July 2024 [64]. Participant recruitment and data collection for aim 2 began in January 2023, and we expect the pilot case series results to be published in 2025. The study is currently in the data collection phase for aim 3, and we expect results from the remaining aims of this study to be published in 2026.

## Discussion

### Anticipated Findings

The goal of this study is to evaluate the effectiveness and feasibility of a school-based, indicated prevention version of the UP-A, which will be branded as the U-PEACE program. U-PEACE will be designed using an iterative approach within Title 1 high schools that primarily serve minoritized youth. To optimize the sustainability of U-PEACE, it will be important to evaluate dissemination and implementation factors throughout the process of designing the intervention [101]. Furthermore, the focus of this study will be to identify and implement warranted modifications within the communities they will ultimately target [102]. Taking this approach will ensure that modifications made are consistent with the community’s needs and will use resources native to the Title 1 school context.

### Study Strengths and Limitations

This study has multiple strengths. First, the use of a mixed qualitative and quantitative design allows for in-depth understanding of the rationale for potential intervention modifications, as well as contextualization of outcomes as relevant hypotheses are tested [103]. Furthermore, the use of an iterative process ensures integration of school community member feedback into the modifications of an existing evidence-based program. The iterative process increases the likelihood that modifications are consistent with the values, goals, and resources within the Title 1 school context and culturally appropriate for the target audience. Finally, the use of RCIs enhances understanding of the potential clinical relevance of U-PEACE when examining early results from the case series. Potential limitations of the study include an insufficient sample size to conduct mediation analyses, which may lead to a restricted range in relevant treatment outcomes. In addition, Title 1 high schools across the country are comprised of diverse community members; while some of the study participants’ experiences may be generalizable to most Title 1 high schools, other identities and experiences (eg, primarily Hispanic or Latine and Afro-Caribbean racial and ethnic identities) may be less generalizable.

### Potential Study Implications

Findings from this study may inform adaptation approaches (eg, specific procedural and content modifications to consider) when implementing existent EBTs into Title 1 high school settings. Specifically, qualitative findings may identify themes related to psychoeducational content that need to be included within mental health interventions for minoritized youth. Second, factors associated with successful implementation of school-based mental health interventions—such as potential professionals providing the intervention timing, and support factors—will be identified. Furthermore, the preliminary cost findings can inform mental health professionals and funding sources of requisite resources for the implementation of similar transdiagnostic programs. Finally, the study will provide information about the acceptability and feasibility of implementing a transdiagnostic indicated prevention program into Title 1 high school communities.

## Appendix

Multimedia Appendix 1 Sample teen interview guide from aim 3 (randomized controlled trial) of the Unified Protocol for Transdiagnostic Treatment of Emotional and Academic Challenges in Education study.

Multimedia Appendix 2 Sample informed consent form for caregivers of participants in aim 3 (randomized controlled trial) of the Unified Protocol for Transdiagnostic Treatment of Emotional and Academic Challenges in Education study (English version).

Multimedia Appendix 3 Sample informed consent form for caregivers of participants in aim 3 (randomized controlled trial) of the Unified Protocol for Transdiagnostic Treatment of Emotional and Academic Challenges in Education study (Spanish version).

Multimedia Appendix 4 SPIRIT (Standard Protocol Items: Recommendations for Interventional Trials) checklist for the Unified Protocol for Transdiagnostic Treatment of Emotional and Academic Challenges in Education study.

Multimedia Appendix 5 Peer review report from the Institute of Education Sciences, United States Department of Education.

## Abbreviations

AAPC: Adolescent Academic Problems Checklist
AMAS-ZABB: Abbreviated Multidimensional Acculturation Scale
ARI: Affective Reactivity Index
BFS: Behavior and Feelings Survey
CAIS-AS: Child Anxiety Impact Scale–Academic Subscale
CBT: cognitive behavioral therapy
CSQ: Client Satisfaction Questionnaire
DIAMOND-KID: Diagnostic Interview for Anxiety, Mood, and OCD-Related Neuropsychiatric Disorders: Child and Adolescent Version
DTS: Distress Tolerance Scale
EBT: evidence-based treatment
EDS: Everyday Discrimination Scale
GAD-7: Generalized Anxiety Disorder 7-item Scale
ICC: intraclass correlation coefficient
PHQ-8: Patient Health Questionnaire-8
PHQ-9: Patient Health Questionnaire-9
RCADS-SF: Revised Children’s Anxiety and Depression Scale–Short Form
RCI: reliable change index
RCT: randomized controlled trial
RQA: rapid qualitative analysis
SAU: service as usual
TASC-R: Therapeutic Alliance Scale for Children−Revised
UP-A: Unified Protocol for Transdiagnostic Treatment of Emotional Disorders in Adolescents
U-PEACE: Unified Protocol for Transdiagnostic Treatment of Emotional and Academic Challenges in Education

## Data Availability

Once data collection and analysis for this study are completed, deidentified data will be available via an open access site.
