# Supplementary material for: An Iteratively Adapted Transdiagnostic Prevention Program for Diverse High School Settings (U-PEACE): Protocol for a Randomized Controlled Trial
Source: JMIR Res Protoc. 2025 Sep 24;14:e74080. doi: 10.2196/74080 (PMC12508673; doi:10.2196/74080)
Supplement: Multimedia Appendix 1 [file resprot_v14i1e74080_app1.pdf]

Informed Consent to Participate in a Research Study

Principal Investigator:

Jill Ehrenreich-May, Ph.D.
Professor and Associate Chair for Graduate Studies
Department of Psychology
University of Miami
P.O. Box 248185
Coral Gables, FL 33124-0751
305-284-6476

Title of Research Study: U-PEACE Randomized Controlled Trial

External Sponsor/Funding Entity: Institute of Education Sciences

IRB Number: 20230796

<u>What is the purpose of this research study?</u>
Your child is being asked to participate in a research study. Researchers at the University of Miami (UM) are interested in evaluating feasibility and effectiveness of a program for high school students with emotional and academic challenges (Unified Protocol for Preventing Emotional and Academic Challenges in Education; U-PEACE) and gaining feedback on that program through interviews.

<u>Who is eligible to participate?</u>
Teens are eligible to participate in this study if (a) they are 13 years or older; (b) they can read and speak in English or Spanish: (c) they can understand the information outlined in the assent form; (d) they are enrolled in a participating Miami-Dade County Public School (M-DCPS) high school; (e) they are seeking additional emotional and/or academic support; and (f) they are or will be consented to receive services through the University of Miami School Health Initiative Clinic.

<u>Why is my child being invited to participate?</u>
Your child is invited to participate in this study because they have indicated they are experiencing emotional challenges during pre-screening or their evaluation at the UM school health initiative (SHI) clinic at their school. This initial evaluation at the UM school health initiative clinic may have indicated that your child may be having a hard time managing strong emotions and is interested in receiving additional support. Miami-Dade County Public School district has reviewed the research study and agreed to participate as a study site.

<u>Is participation voluntary?</u>
Your consent and the participation of your child in this study are voluntary. Your child does not have to be in this study if they do not want to, and they can leave the study at any time. You or your child will not lose any services, benefits, or rights you normally have if you or your child choose not to be in the study or if your child leaves the study early. Your child's participation or absence of participation in this study will also not affect your or your child's involvement in other UM studies. Before deciding whether to allow your child to participate in this research study, please read this consent form carefully and discuss any questions you have with the study team.

<u>How long will the participation in this study last?</u>
Participation in this study will be for approximately 13 weeks.

What are the costs for participating in this study?
You and your child are not responsible for any costs of this program.

What will my child be asked to do?
If you and your child consent to your child participating in the study, and your child is eligible to participate, your child will be randomly assigned (by chance, like the flip of a coin) to either be enrolled in the U-PEACE program or receive SHI clinic services.

U-PEACE Program:
The U-PEACE program group will include about six other students from their high school (or other high schools, if online). Your child's school will have professionals trained in delivering U-PEACE to high school students. This program will involve about 13 group sessions, with each group session lasting approximately 40-50 minutes. The program sessions will be held about 2 times per week during your child's school day (e.g., lunch time) if the group meets in person, or at a pre-scheduled time every week if the group meets online via Zoom. If the group is held in person, your child will be offered lunch during the group meetings. If your child is not able to attend the group session in a given week, a member of the U-PEACE team may attempt to meet with your child individually for a make-up U-PEACE session before the next group session. Your child's make-up sessions may occur during lunch period, after school, during elective courses, or online via Zoom. The group and make-up sessions will be facilitated by professionals on the U-PEACE team who are trained to deliver the program.

The U-PEACE group leaders use approaches that are supported by research to help teenagers cope with their emotions and perform better academically. In this program, teens will learn how to better understand their emotional experiences and use their understanding to embrace agency in challenging situations. Teens will develop skills to better regulate their emotions by increasing awareness of what is happening around them and choose how they respond. Skill-building exercises may include mindfulness practices, flexible thinking, behavioral activation, problem-solving, and exposure activities. Exposure activities may involve you doing things that can cause distress or doing things that you usually avoid. The point of exposure in sessions is to practice, refine, and improve skills in real-life situations.

Group leaders may do exposures or behavioral-activation work in the UM School Health Initiative (SHI) clinic on your child's campus, in other places on campus, off campus, or virtually. These sessions may also involve other M-DCPS staff if it would be helpful to do so. Group leaders will adhere to the same legal and ethical guidelines during sessions outside of the research clinic as they would follow within the research clinic. It is more difficult to protect confidentiality outside of the confines of the research clinic and/or when additional individuals are involved in exposure or activation procedures but assigned group leaders will do their best to maintain participant confidentiality. The University of Miami, U-PEACE staff, and/or M-DCPS staff are not responsible for any accident or injury that may occur outside the UM SHI clinic.

During the program sessions, a member of the research team may be present to take notes about the program's progress. The sessions may also be video- and/or audio-recorded to be reviewed by study personnel to ensure that the group leaders are delivering the program correctly. The notes and recordings will be stored in locked drawers in locked offices at the University of Miami and on university-approved computers and servers, and they will be reviewed only by members of our study team. Notes will not include any names of participants in the program.

After the program has ended, your child may be selected to participate in an individual interview asking for their feedback on U-PEACE. This interview is expected to take approximately 30 minutes. To

accurately capture what is said, the interview will be audio recorded. Your child may ask to pause or stop the recording at any time. Your child can choose how much or how little they want to speak during the interview. Your child may choose to leave the interview at any time.

SHI Services:
If your child is assigned to receive the SHI services, they will be able to continue to access any services available through the UM SHI clinic (e.g., medical care, health education, case management, reproductive health services) determined to be needed. Any psychotherapy or mental health services (e.g., counseling) your child may receive during your participation in this study will be monitored.

Your child will also receive lunch and be asked to complete brief study questionnaires 2 times a week. A study team member or school mental health providers may assess and provide linkage to UM SHI services needed and monitor for clinical deterioration. If it is determined that your child is experiencing deterioration, additional mental health services or appropriate referrals will be offered following consultation with Dr. Ehrenreich-May, Dr. Pulgaron, and/or Dr. Gwynn, who are the investigators of the study. When your child's participation in this study ends, your child will be offered participation in the subsequent U-PEACE program group.

Questionnaires and Tasks:
As part of the study, your child will complete questionnaires and assessments with a study team member at four different times: at the beginning of the study, about 4 weeks into the study, about 8 weeks into the study, and about 11 weeks into the study. Your child may also be asked to provide their school report that shows their school grades. Additionally, you and/or high school staff (e.g., teachers) may complete questionnaires about your child. Questionnaires may inquire about your child, their emotions, academic functioning, and stressors your child may be experiencing. Questionnaires will be given to you, your child, and/or high school staff to complete using pen and paper or online. We will also ask your child to identify one teacher who your child thinks knows them well to complete one questionnaire.

Release of Information:
To participate in the research study, your child must be consented to receive services from the University of Miami SHI clinic. Once your child has been consented to receive services from the UM SHI clinic, the study team may access your child's clinic records to obtain information about the services your child uses at the clinic. The UM SHI clinic may ask you and/or your child to sign an additional form giving the clinic permission to release that information to the study team.

What are the risks of participating in this study?
Your child may feel uncomfortable answering some of the study questions and/or being taped. Your child may always choose not to answer a question that makes them feel uncomfortable. There are no physical risks associated with participating in this study.

What are the benefits of participating in this study?
While there is no guarantee of benefits, your child may feel better or improve academically and emotionally.

Will I and/or my child be compensated for participating in this study?
Your child will be compensated up to $225 in this study. Payments will be delivered in the form of gift cards and will occur at each of the questionnaire/assessment completion time point: $50 for completion of the first set of questionnaires and assessments, $25 for completion of the second set of questionnaires, $50 for completion of the third set of questionnaires, and $50 for completion of the fourth set of questionnaires. Additionally, if your child is selected to complete an interview asking for their U-PEACE

program feedback at the end of the program, your child will be given $50 for completing the interview. Your child may be given a meal at each U-PEACE group session and for completing the feedback interview. You, as a caregiver, may also receive up to $40 in this study in the form of gift cards: $10 for more than 50% completion of the questionnaires about your child at each questionnaire/assessment time point. If you are selected to complete an interview asking for your feedback on the U-PEACE program, you will be given an additional $50 for completing the interview.

What alternative procedures are available to me and my child?
If you decide not to allow your child to join this study, neither you nor your child's care at the UM SHI clinic will be affected, and you and your child can still receive the standard services available at the UM SHI clinic at your child's school. You can ask the healthcare workers at the UM SHI about other services available that may be of help to your child.

Confidentiality
The information you will share with us if you participate in this study will be kept completely confidential, or private, to the full extent of the law. Your name will never be used in the results of this study. We use identification numbers/initials instead of names.

Please be advised that although the researchers will take every precaution to maintain confidentiality of the data, the nature of U-PEACE groups prevents the researchers from guaranteeing confidentiality. The researchers will remind all participants of U-PEACE to respect the privacy of their fellow participants and not repeat what is said in the U-PEACE groups to others. If any interview takes place on Zoom, you and/or your child will be asked to change your name on Zoom before any recording starts and to not use any names during the interview.

Audio-video recordings and study data will be securely stored on password-protected computers in locked research offices, locked file-cabinets in locked offices in the Psychology Building on the Coral Gables Campus, on the Psychology Department's secure server, or on a UM-supported cloud software (e.g., RedCap, SharePoint) accessible only to authorized study personnel who have been trained to protect your privacy.

After the interviews, recordings will be typed using a transcription service (e.g., Datagain Transcription), and the typed transcription will also be kept in a secured location. Only the people who are directly involved with the study will be able to view your information, listen to recordings, or read the typed version of recordings.

No information from this study will be shared outside of the people/parties listed above without your written consent.

Limits of confidentiality
In accordance with Florida State laws and the American Psychological Association's Ethics Code, confidentiality may be waived without your consent under the following conditions: 1) If you or your child are deemed in danger of harming yourself or others, 2) If the group leaders or study team believes that a child is being neglected or abused, 3) If the group leaders or study team believes that an elderly person is being abused or neglected, 4) If the U-PEACE team, its program facilitators, supervisors, or the University of Miami receives a valid court order to release your child's records, 5) If you file an ethical or legal complaint against the study team, the group leaders, their supervisor, the SHI, or the University of Miami. The U.S Department of Health and Human Services (DHHS) may request to review and get copies of your child's records.

<u>Contact over the Internet and via text</u>
Usually, research team members only talk to teen participants in person and/or in the presence of a caregiver. Since this study is occurring during school hours, some teens may prefer independently communicating with us over the Internet and/or via text. Since communication over the Internet and via text is not secure and may not remain confidential, we need your permission to communicate with your child using these means. This is voluntary and will not affect your child's participation in the study.

If you would like to take advantage of these options, please initial your choice below:

Permission to Contact You (Please put your <u>initials</u> next to <u>ONE</u> of the below)

______ I **give** staff permission to contact me over the internet and via text.

______ I **do not give** staff permission to contact me over the internet and via text.

Permission to Contact Your Child (Please put your <u>initials</u> next to <u>ONE</u> of the below)

______ I **give** staff permission to contact my child over the internet and via text.

______ I **do not give** staff permission to contact my child over the internet and via text.

<u>What if I decide to stop participating in the study?</u>
- You and/or your child can agree to be in the study now and can change your mind later.
- If you and/or your child wish for your child to stop being in the study, tell the study staff right away.
- Leaving this study early will not stop your child from getting regular medical care and will not affect your relationship with the UM SHI clinic or with University of Miami.

If you decide to withdraw, we ask that you let us know by calling Dr. Jill Ehrenreich-May, the Principal Investigator of the study, or by sending a written notice (contact information is on the first page). If you withdraw from the study, data that has already been collected will still be used for the study, unless you specify otherwise; no further information will be collected from you after study withdrawal.

<u>Can someone else make my child stop participating in this study?</u>
You and/or your child may want your child to be taken out of the study if:
- Staying in the study would be harmful or unsafe.
- The study is cancelled or has ended.
- There may be other reasons that we do not know at this time to take you and/or your child out of the study.

<u>Is there anything else I need to consider?</u>
The Principal Investigator on this study has a financial interest in this project. The U-PEACE program is an adaptation of the Unified Protocol for Transdiagnostic Treatment of Emotional Disorders in Adolescents (UP-A). Dr. Jill Ehrenreich-May is the first author of the therapist guide and workbooks for the UP-A, and receives royalties from these publications. She also receives payments for UP-A clinical trainings, consultation and implementation support services.

A description of this study will be available on http://www.ClinicalTrials.gov. This website will not include information that can identify you nor your child. At most, the website will include a summary of the study results. You can search this website at any time.

What if I have questions?

The Principal Investigator and all study staff are willing to answer any questions you and your child may have about the research. You and your child are encouraged to ask questions before deciding whether to take part. You and your child are also encouraged to ask questions during the study. If you and/or your child have questions, complaints, or concerns about the research, you may call Dr. Jill Ehrenreich-May, the Principal Investigator (305-284-6476).

You may also call a coordinator at the Institutional Review Board at (305) 243-3195, if you want to talk to someone who is not a member of the research team in order to pass along any suggestions, complaints, concerns, or compliments about your involvement in the research, or to ask general questions or obtain information about participation in clinical research studies. Please do not call the IRB number for medical related issues or to schedule or cancel an appointment.

PARTICIPATION AGREEMENT:

I have read the information in this consent form and agree to allow my child to participate in this research study. I have had the chance to ask questions about this research, and they have been answered. I can have a copy of this form to keep, whether I allow my child to participate or not.

Your signature on this line indicates your agreement to permit the named child to take part in this research.

____

Printed Name of Child

____

Printed Name of Parent/Legally Authorized Representative

____ ______________________

Signature of Parent/Legally Authorized Representative Date

PARTICIPATION REFUSAL:

I have read the information in this consent form and do not agree to allow my child to participate in this research study. I have had the chance to ask questions about this research, and they have been answered. I can have a copy of this form to keep, whether I allow my child to participate or not.

Your signature on this line indicates your refusal to permit the named child to take part in this research.

____

Printed Name of Child

____
Printed Name of Parent/Legally Authorized Representative

____ ____________________
Signature of Parent/Legally Authorized Representative Date

____
Printed Name of Person Obtaining Consent

____ ____________________
Signature of Person Obtaining Consent Date

To be completed by research staff:

- ☐ Assent obtained
- ☐ Assent not obtained due to child refusal
