# Supplementary material for: An Iteratively Adapted Transdiagnostic Prevention Program for Diverse High School Settings (U-PEACE): Protocol for a Randomized Controlled Trial
Source: JMIR Res Protoc. 2025 Sep 24;14:e74080. doi: 10.2196/74080 (PMC12508673; doi:10.2196/74080)
Supplement: Multimedia Appendix 2 [file resprot_v14i1e74080_app2.pdf]

**Consentimiento informado para participar en un estudio de investigación**

**Investigador Principal:**

Jill Ehrenreich-May, Ph.D.
Profesor y Cátedra Asociada de Estudios de Posgrado
Departamento de Psicología
Universidad de Miami
Apartado de correos 248185
Coral Gables, FL 33124-0751
305-284-6476

**Título del estudio de investigación:** U-PEACE Randomized Controlled Trial

**Patrocinador externo/entidad financiadora:** Instituto de Ciencias de la Educación

**Número IRB:** 20230796

**¿Cuál es el propósito de este estudio de investigación?**

Se le pide a su hijo que participe en un estudio de investigación. Investigadores de la Universidad de Miami (UM) están interesados en evaluar la viabilidad y efectividad de un programa para estudiantes de secundaria con dificultades emocionales y académicos (Protocolo Unificado para la Prevención de Dificultades Emocionales y Académicos en la Educación; U-PEACE) y obtener comentarios sobre ese programa a través de entrevistas.

**¿Quién es elegible para participar?**

Los adolescentes son elegibles para participar en este estudio si (a) tienen 13 años o más; (b) pueden leer y hablar en inglés o español: (c) pueden entender la información descrita en el formulario de consentimiento; (d) están inscritos en una escuela secundaria participante de las Escuelas Públicas del Condado Miami-Dade (M-DCPS); (e) están buscando apoyo emocional y/o académico adicional; y (f) están o serán consentidos para recibir servicios a través de la Clínica de la Iniciativa de Salud Escolar de la Universidad de Miami.

**¿Por qué se invita a mi hijo a participar?**

Su hijo está invitado a participar en este estudio porque ha indicado que está experimentando dificultades emocionales durante la preselección o su evaluación en la clínica de la iniciativa de salud escolar (SHI) de UM en su escuela. Esta evaluación inicial en la clínica de la iniciativa de salud escolar de UM puede haber indicado que su hijo puede estar teniendo dificultades para manejar las emociones fuertes y está interesado en recibir apoyo adicional. El Distrito de Escuelas Públicas del Condado Miami-Dade ha revisado el estudio de investigación y acordó participar como un sitio de estudio.

**¿La participación es voluntaria?**

Su consentimiento y la participación de su hijo en este estudio son voluntarios. Su hijo no tiene que estar en este estudio si no lo desea, y puede abandonar el estudio en cualquier momento. Usted o su hijo no perderán ningún servicio, beneficio o derecho que normalmente tienen si usted o su hijo deciden no participar en el estudio o si su hijo abandona el estudio antes de tiempo. La participación o ausencia de participación de su hijo en este estudio tampoco afectará su participación o la de su hijo en otros estudios de UM. Antes de decidir si permite que su hijo participe en este estudio de investigación, lea atentamente este formulario de consentimiento y discuta cualquier pregunta que tenga con el equipo del estudio.

**¿Cuánto tiempo durará la participación en este estudio?**
La participación en este estudio será de aproximadamente 13 semanas.

**¿Cuáles son los costos de participar en este estudio?**
Usted y su hijo no son responsables de ningún costo de este programa.

**¿Qué se le pedirá a mi hijo que haga?**
Si usted y su hijo dan su consentimiento para que su hijo participe en el estudio, y su hijo es elegible para participar, su hijo será asignado al azar (por casualidad, como el lanzamiento de una moneda) para inscribirse en el programa U-PEACE o recibir servicios clínicos de SHI.

**Programa U-PEACE:**
El grupo del programa U-PEACE incluirá a otros seis estudiantes de su escuela secundaria (u otras escuelas secundarias, si están en línea). La escuela de su hijo tendrá profesionales capacitados en la entrega de U-PEACE a los estudiantes de secundaria. Este programa incluirá alrededor de 13 sesiones grupales, con una duración aproximada de 40-50 minutos. Las sesiones del programa se llevarán a cabo aproximadamente 2 veces por semana durante el día escolar de su hijo (por ejemplo, a la hora del almuerzo) si el grupo se reúne en persona, o a una hora programada previamente cada semana si el grupo se reúne en línea a través de Zoom. Si el grupo se lleva a cabo en persona, a su hijo se le ofrecerá el almuerzo durante las reuniones grupales.Si su hijo  no puede asistir a la sesión grupal en una semana determinada, un miembro del equipo de U-PEACE puede intentar reunirse con su hijo individualmente para una sesión de recuperación de U-PEACE antes de la próxima sesión grupal. Las sesiones de recuperación de su hijo pueden ocurrir durante el período de almuerzo, después de la escuela, durante cursos electivos o en línea a través de Zoom. Las sesiones grupales y de recuperación serán facilitadas por profesionales del equipo de U-PEACE que están capacitados para impartir el programa.

Los líderes del grupo U-PEACE utilizan enfoques respaldados por la investigación para ayudar a los adolescentes a lidiar con sus emociones y desempeñarse mejor académicamente. En este programa, los adolescentes aprenderán cómo comprender mejor sus experiencias emocionales y usar su comprensión para abrazar el albedrío en situaciones difíciles. Los adolescentes desarrollarán habilidades para regular mejor sus emociones al aumentar la conciencia de lo que está sucediendo a su alrededor y elegir cómo responden. Los ejercicios de desarrollo de habilidades pueden incluir prácticas de atención plena, pensamiento flexible, activación conductual, resolución de problemas y actividades de exposición. Las actividades de exposición pueden implicar que usted haga cosas que pueden causar angustia o hacer cosas que generalmente evita. El punto de exposición en las sesiones es practicar, refinar y mejorar las habilidades en situaciones de la vida real.

Los líderes de grupo pueden hacer exposiciones o trabajo de activación conductual en la clínica de la Iniciativa de Salud Escolar (SHI) de la UM en el campus de su hijo, en otros lugares del campus, fuera del campus o virtualmente. Estas sesiones también pueden involucrar a otro personal de las M-DCPS si fuera útil hacerlo. Los líderes de grupo se adherirán a las mismas pautas legales y éticas durante las sesiones fuera de la clínica de investigación que seguirían dentro de la clínica de investigación. Es más difícil proteger la confidencialidad fuera de los límites de la clínica de investigación y / o cuando individuos adicionales están involucrados en procedimientos de exposición o activación, pero los líderes de grupo asignados harán todo lo posible para mantener la confidencialidad de los participantes. El personal de la Universidad de Miami, U-PEACE y/o el personal de las M-DCPS no son responsables de ningún accidente o lesión que pueda ocurrir fuera de la clínica UM SHI.

Durante las sesiones del programa, un miembro del equipo de investigación puede estar presente para tomar notas sobre el progreso del programa. Las sesiones también pueden ser grabadas en video y / o

audio para ser revisadas por el personal del estudio para garantizar que los líderes del grupo estén entregando el programa correctamente. Las notas y grabaciones se almacenarán en cajones cerrados con llave en oficinas cerradas en la Universidad de Miami y en computadoras y servidores aprobados por la universidad, y serán revisados solo por miembros de nuestro equipo de estudio. Las notas no incluirán ningún nombre de los participantes en el programa.

Después de que el programa haya terminado, su hijo puede ser seleccionado para participar en una entrevista individual solicitando sus comentarios sobre U-PEACE. Se espera que esta entrevista dure aproximadamente 30 minutos. Para capturar con precisión lo que se dice, la entrevista será grabada en audio. Su hijo puede pedirle que detenga o detenga la grabación en cualquier momento. Su hijo puede elegir cuánto o qué tan poco quiere hablar durante la entrevista. Su hijo puede optar por abandonar la entrevista en cualquier momento.

**Servicios SHI:**
Si a su hijo se le asigna recibir los servicios de SHI, podrá continuar accediendo a cualquier servicio disponible a través de la clínica UM SHI (por ejemplo, atención médica, educación para la salud, administración de casos, servicios de salud reproductiva) que se determine que son necesarios. Cualquier psicoterapia o servicios de salud mental (p. ej., asesoramiento) que su hijo pueda recibir durante su participación en este estudio será monitoreado.

Su hijo también recibirá almuerzo y se le pedirá que complete breves cuestionarios de estudio 2 veces a la semana. Un miembro del equipo del estudio o proveedores de salud mental de la escuela pueden evaluar y proporcionar vinculación a los servicios de UM SHI necesarios y monitorear el deterioro clínico. Si se determina que su hijo está experimentando deterioro, se le ofrecerán servicios adicionales de salud mental o referencias apropiadas después de consultar con el Dr. Ehrenreich-May, el Dr. Pulgaron y / o el Dr. Gwynn, quienes son los investigadores del estudio. Cuando termine la participación de su hijo en este estudio, se le ofrecerá su participación en el grupo posterior del programa U-PEACE.

**Cuestionarios y tareas:**
Como parte del estudio, su hijo completará cuestionarios y evaluaciones con un miembro del equipo del estudio en cuatro momentos diferentes: al comienzo del estudio, aproximadamente 4 semanas después del estudio, aproximadamente 8 semanas después del estudio y aproximadamente 11 semanas después del estudio. También se le puede pedir a su hijo que proporcione su informe escolar que muestre sus calificaciones escolares. Además, usted y/o el personal de la escuela secundaria (p. ej., maestros) pueden completar cuestionarios sobre su hijo. Los cuestionarios pueden preguntar sobre su hijo, sus emociones, funcionamiento académico y factores estresantes que su hijo puede estar experimentando. Se le entregarán cuestionarios a usted, a su hijo y / o al personal de la escuela secundaria para que los complete con lápiz y papel o en línea. También le pediremos a su hijo que identifique a un maestro que su hijo piense que lo conoce bien para completar un cuestionario.

**Divulgación de información:**
Para participar en el estudio de investigación, su hijo debe recibir su consentimiento para recibir servicios de la clínica SHI de la Universidad de Miami. Una vez que su hijo haya recibido su consentimiento para recibir servicios de la clínica UM SHI, el equipo del estudio puede acceder a los registros clínicos de su hijo para obtener información sobre los servicios que su hijo usa en la clínica. La clínica UM SHI puede pedirle a usted y/o a su hijo que firmen un formulario adicional que le dé permiso a la clínica para divulgar esa información al equipo del estudio.

**¿Cuáles son los riesgos de participar en este estudio?**

Su hijo puede sentirse incómodo respondiendo algunas de las preguntas del estudio y/o siendo grabado. Su hijo siempre puede optar por no responder una pregunta que lo haga sentir incómodo. No hay riesgos físicos asociados con la participación en este estudio.

**¿Cuáles son los beneficios de participar en este estudio?**
Si bien no hay garantía de beneficios, su hijo puede sentirse mejor o mejorar académica y emocionalmente.

**¿Se nos compensará a mí y/o a mi hijo por participar en este estudio?**
Su hijo recibirá una compensación de hasta $225 en este estudio. Los pagos se entregarán en forma de tarjetas de regalo y se realizarán en cada uno de los puntos de tiempo de finalización del cuestionario / evaluación: $ 50 para completar el primer conjunto de cuestionarios y evaluaciones, $ 25 para completar el segundo conjunto de cuestionarios, $ 50 para completar el tercer conjunto de cuestionarios y $ 50 para completar el cuarto conjunto de cuestionarios. Además, si su hijo es seleccionado para completar una entrevista solicitando sus comentarios sobre el programa U-PEACE al final del programa, su hijo recibirá $50 por completar la entrevista. A su hijo se le puede dar una comida en cada sesión grupal de U-PEACE y para completar la entrevista de retroalimentación. Usted, como cuidador, también puede recibir hasta $40 en este estudio en forma de tarjetas de regalo: $10 por completar más del 50% de los cuestionarios sobre su hijo en cada punto de tiempo del cuestionario/evaluación. Si es seleccionado para completar una entrevista solicitando sus comentarios sobre el programa U-PEACE, se le darán $ 50 adicionales por completar la entrevista.

**¿Qué procedimientos alternativos están disponibles para mí y mi hijo?**
Si decide no permitir que su hijo se una a este estudio, ni usted ni la atención de su hijo en la clínica UM SHI se verán afectadas, y usted y su hijo aún pueden recibir los servicios estándar disponibles en la clínica UM SHI en la escuela de su hijo. Puede preguntar a los trabajadores de atención médica en UM SHI sobre otros servicios disponibles que pueden ser de ayuda para su hijo.

**Confidencialidad**
La información que compartirá con nosotros si participa en este estudio se mantendrá completamente confidencial o privada, en toda la extensión de la ley. Su nombre nunca será utilizado en los resultados de este estudio. Utilizamos números/iniciales de identificación en lugar de nombres.

Tenga en cuenta que aunque los investigadores tomarán todas las precauciones para mantener la confidencialidad de los datos, la naturaleza de los grupos U-PEACE impide que los investigadores garanticen la confidencialidad. Los investigadores recordarán a todos los participantes de U-PEACE que respeten la privacidad de sus compañeros participantes y no repitan lo que se dice en los grupos de U-PEACE a los demás. Si alguna entrevista se lleva a cabo en Zoom, se le pedirá a usted y / o a su hijo que cambien su nombre en Zoom antes de que comience cualquier grabación y que no usen ningún nombre durante la entrevista.

Las grabaciones de audio y video y los datos del estudio se almacenarán de forma segura en computadoras protegidas con contraseña en oficinas de investigación cerradas, archivadores cerrados en oficinas cerradas en el Edificio de Psicología en el Campus de Coral Gables, en el servidor seguro del Departamento de Psicología o en un software en la nube compatible con UM (por ejemplo, RedCap, SharePoint) accesible solo para el personal autorizado del estudio que haya sido capacitado para proteger su privacidad.

Después de las entrevistas, las grabaciones se escribirán utilizando un servicio de transcripción (por ejemplo, Datagain Transcription), y la transcripción mecanografiada también se mantendrá en un lugar

seguro. Solo las personas que están directamente involucradas con el estudio podrán ver su información, escuchar grabaciones o leer la versión mecanografiada de las grabaciones.

Ninguna información de este estudio se compartirá fuera de las personas / partes mencionadas anteriormente sin su consentimiento por escrito.

**Límites de la confidencialidad**

De acuerdo con las leyes del estado de Florida y el Código de Ética de la Asociación Americana de Psicología, se puede renunciar a la confidencialidad sin su consentimiento bajo las siguientes condiciones: 1) Si se considera que usted o su hijo están en peligro de lastimarse a sí mismo o a otros, 2) Si los líderes del grupo o el equipo del estudio creen que un niño está siendo descuidado o abusado, 3) Si los líderes del grupo o el equipo de estudio creen que una persona mayor está siendo abusada o descuidada, 4) Si el equipo de U-PEACE, sus facilitadores del programa, supervisores o la Universidad de Miami reciben una orden judicial válida para divulgar los registros de su hijo, 5) Si presenta una queja ética o legal contra el equipo de estudio, los líderes del grupo, su supervisor, el SHI, o la Universidad de Miami. El Departamento de Salud y Servicios Humanos de los Estados Unidos (DHHS, por sus siglas en inglés) puede solicitar revisar y obtener copias de los registros de su hijo.

**Contacto a través de Internet y vía texto**

Por lo general, los miembros del equipo de investigación solo hablan con los participantes adolescentes en persona y / o en presencia de un cuidador. Dado que este estudio se realiza durante el horario escolar, algunos adolescentes pueden preferir comunicarse independientemente con nosotros a través de Internet y / o por mensaje de texto. Dado que la comunicación a través de Internet y por mensaje de texto no es segura y puede no permanecer confidencial, necesitamos su permiso para comunicarnos con su hijo utilizando estos medios. Esto es voluntario y no afectará la participación de su hijo en el estudio.

Si desea aprovechar estas opciones, inicie su elección a continuación:

**Permiso para contactarlo (Por favor, ponga sus iniciales junto a UNA de las siguientes)**

______ **Doy** permiso al personal para contactarme a través de Internet y por mensaje de texto.

______ **No doy** permiso al personal para contactarme a través de Internet y por mensaje de texto.

**Permiso para contactar a su hijo (por favor, ponga sus iniciales al lado de UNA de las siguientes)**

______ **Doy** permiso al personal para contactar a mi hijo a través de Internet y por mensaje de texto.

______ **No doy** permiso al personal para contactar a mi hijo a través de Internet y por mensaje de texto.

**¿Qué pasa si decido dejar de participar en el estudio?**

- Usted y/o su hijo pueden aceptar participar en el estudio ahora y pueden cambiar de opinión más adelante.
- Si usted y/o su hijo desean que su hijo deje de participar en el estudio, informe al personal del estudio de inmediato.
- Abandonar este estudio antes de tiempo no impedirá que su hijo reciba atención médica regular y no afectará su relación con la clínica UM SHI o con la Universidad de Miami.

Si decide retirarse, le pedimos que nos lo haga saber llamando a la Dra. Jill Ehrenreich-May, la investigadora principal del estudio, o enviando un aviso por escrito (la información de contacto se encuentra en la primera página). Si se retira del estudio, los datos que ya se han recopilado se seguirán utilizando para el estudio, a menos que especifique lo contrario; No se recopilará más información de usted después de retirar el estudio.

**¿Puede alguien más hacer que mi hijo deje de participar en este estudio?**
Usted y/o su hijo pueden querer que su hijo sea sacado del estudio si:

- Permanecer en el estudio sería perjudicial o inseguro.
- El estudio se cancela o ha finalizado.
- Puede haber otras razones que no sepamos en este momento para sacarlo a usted y/o a su hijo del estudio.

**¿Hay algo más que deba considerar?**
El investigador principal de este estudio tiene un interés financiero en este proyecto. El programa U-PEACE es una adaptación del Protocolo Unificado para el Tratamiento Transdiagnóstico de los Trastornos Emocionales en Adolescentes (UP-A). Jill Ehrenreich-May es la primera autora de la guía de terapeutas y libros de trabajo para la UP-A, y recibe regalías de estas publicaciones. También recibe pagos por capacitaciones clínicas de UP-A, consultoría y servicios de apoyo a la implementación.

Una descripción de este estudio estará disponible en http://www.ClinicalTrials.gov. Este sitio web no incluirá información que pueda identificarlo a usted ni a su hijo. A lo sumo, el sitio web incluirá un resumen de los resultados del estudio. Puede buscar en este sitio web en cualquier momento.

**¿Qué pasa si tengo preguntas?**
El investigador principal y todo el personal del estudio están dispuestos a responder cualquier pregunta que usted y su hijo puedan tener sobre la investigación. Se les anima a usted y a su hijo a hacer preguntas antes de decidir si participarán. También se alienta a usted y a su hijo a hacer preguntas durante el estudio. Si usted y/o su hijo tienen preguntas, quejas o inquietudes sobre la investigación, puede llamar a la Dra. Jill Ehrenreich-May, la investigadora principal (305-284-6476).

También puede llamar a un coordinador de la Junta de Revisión Institucional al (305) 243-3195, si desea hablar con alguien que no sea miembro del equipo de investigación para transmitir cualquier sugerencia, queja, inquietud o cumplido sobre su participación en la investigación, o para hacer preguntas generales u obtener información sobre la participación en estudios de investigación clínica. No llame al número del IRB para problemas médicos relacionados o para programar o cancelar una cita.

**ACUERDO DE PARTICIPACIÓN:**
He leído la información en este formulario de consentimiento y acepto permitir que mi hijo participe en este estudio de investigación. He tenido la oportunidad de hacer preguntas sobre esta investigación, y han sido respondidas. Puedo tener una copia de este formulario para conservar, ya sea que permita que mi hijo participe o no.

Su firma en esta línea indica su acuerdo para permitir que el niño nombrado participe en esta investigación.

________________________________________

Nombre impreso del niño

______________________________________________
Nombre impreso del padre/representante legalmente autorizado

__________________________________________ ____________________
Firma del padre/representante legalmente autorizado Fecha

**RECHAZO DE LA PARTICIPACIÓN:**

He leído la información en este formulario de consentimiento y no acepto permitir que mi hijo participe en este estudio de investigación. He tenido la oportunidad de hacer preguntas sobre esta investigación, y han sido respondidas. Puedo tener una copia de este formulario para conservar, ya sea que permita que mi hijo participe o no.

Su firma en esta línea indica su negativa a permitir que el niño nombrado participe en esta investigación.

_____________________________________
Nombre impreso del niño

______________________________________________
Nombre impreso del padre/representante legalmente autorizado

__________________________________________ ____________________
Firma del padre/representante legalmente autorizado Fecha

_________________________________
Nombre impreso de la persona que obtiene el consentimiento

__________________________________________ ____________________
Firma de la persona que obtiene el consentimiento Fecha

A cumplimentar por el personal investigador:

- ☐ Dictamen conforme obtenido
- ☐ Asentimiento no obtenido debido a la negativa del niño
