# Supplementary material for: An Iteratively Adapted Transdiagnostic Prevention Program for Diverse High School Settings (U-PEACE): Protocol for a Randomized Controlled Trial
Source: JMIR Res Protoc. 2025 Sep 24;14:e74080. doi: 10.2196/74080 (PMC12508673; doi:10.2196/74080)
Supplement: Multimedia Appendix 3 [file resprot_v14i1e74080_app3.pdf]

Last Update: 1/13/25

U-PEACE Post-RCT Interview Question Guide(s)

Welcome & Introduction:

Thank you for taking the time to participate in this session. The purpose of this interview session is to understand your experiences with the U-PEACE program and get your feedback on how we can improve the program for teens who have a hard time managing strong emotions. Your input as a *[teen, caregiver, mental health provider, coach, school administrator]* is especially valuable to us. All the feedback you provide today will be used to make the program better.

Overview of Interview:

This interview session will last about 45 minutes. During this session, we will ask you some general questions about your overall thoughts and opinions regarding the program. After the session, you will receive a *$50* gift card for participating. We will also plan for you to receive a free meal during the interview as a thank you
Interview Guidelines:
There are a few guidelines for the interview:

1. We will be recording this interview because we do not want to miss any of your comments/feedback.
2. You have the right to stop this interview at any time and you can skip any questions that you do not want to answer. Please ask questions if you are confused about any questions.
3. (If group interview) All of your opinions count, and it is okay to disagree. We ask that you be respectful of one another. We would like to hear from each of you, but only one at a time.
4. Please share all information with us. We are interested in everything you have to say – including both the good and the bad (and the neutral). You may have more thoughts on some parts of the program than on others.
5. I will be guiding this discussion. I will make every effort to keep the discussion focused. If too much time is being spent on one question, I may move the conversation along so we can cover all the questions. (If group: All that said, you do not have to speak to me directly. You may direct your comments to other members of the group.) The idea is to have a fluid discussion.

Recording:

(*For caregiver interview*: Per your informed consent today,) We will now begin recording.

****BEGIN RECORDING NOW****

Last Update: 1/13/25

Interview Questions (ADOLESCENTS)

SECTION 1: Specific Feedback about U-PEACE (~15-20 minutes)

Let's start with your feedback about U-PEACE.

1. Tell me about your experience in the U-PEACE program.
   a. *Probe:* What parts of the program did you like or find most helpful?
   b. *Probe:* What parts of the program did you find least helpful or were your least favorite?
2. A lot of times, programs like U-PEACE are delivered in non-school settings like the clinic. What was it like to participate in this type of program at your school?
   a. *Probe:* What, if any, were some facilitators or things that helped you participate in a program like this at your school?
   b. *Probe:* What, if any, were some challenges or barriers that might have kept you from participating in the program at your school?
3. What, if any, impact did U-PEACE have on you?
   a. *Probe:* How, if at all, did U-PEACE affect your academic performance? Or how you think about your academic performance? How, if at all, did you practice what you learned in U-PEACE?
   a. *Probe:* How, if at all, did you use exposure in school? How often?
   b. *Probe:* How, if at all, did you use exposure outside of school? How often?
   c. *Probe:* How, if at all, did you use opposite action at school? How often?
   d. *Probe:* How, if at all, did you use opposite action at home? How often?
   e. *Probe:* How, if at all, did you use opposite action in other contexts? What contexts? How often?
   f. *Probe:* What other sessions or content from U-PEACE did you find particularly helpful?
   g. Probe: What was not helpful?
4. What did you like about the U-PEACE program's support materials (e.g., workbook, homework, exposure/activation practice logs, session summaries, caregiver materials and if they were given to the caregivers)? What did you dislike about the support materials?

SECTION 2: Using U-PEACE with Diverse Clients and Cultural Modifications (~5 minutes)

1. How well do you think the U-PEACE program works with students who go to your school? Why?
2. How well do you think U-PEACE works with students from diverse racial and ethnic cultures/backgrounds or with multiple life experiences? Why?
3. If you were the developer of U-PEACE, how would you design it?

SECTION 3: Logistics of U-PEACE (~15-20 minutes)

1. What did you think about meeting for U-PEACE during lunchtime at school?
   a. *Probe:* What did you like about having the group during lunchtime?
   b. *Probe:* What prevented you from attending during lunchtime?
   c. *Probe:* If you had to suggest a generally good time for U-PEACE, other than lunchtime, when would it be?
2. What motivated you to attend U-PEACE sessions?

   a. *Probe:* Would you have still come to U-PEACE sessions even if there was no lunch?
3. What did you think about the group format of U-PEACE, with about 5-6 other students?
   a. *Probe:* What did you like about doing U-PEACE sessions in a group? Why?
   b. *Probe:* What did you not like about having the sessions in a group? Why?
4. How many sessions of U-PEACE would you have liked per week? Overall?
   a. *Probe*: Would you still attend U-PEACE sessions even during testing season?
5. If you have missed a session (or sessions), what helped you catch up on what you missed?
   a. *Probe*: Did you find meeting with your coach [COACH NAME] helpful? Why or why not?
   b. *Probe*: How could we have helped you better to catch up on what you missed?
   c. *Probe:* Did you find reviewing the session summaries helpful when you missed a session? Why or why not?
6. Describe your experiences with the assigned coach, [NAME OF COACH]:
   a. *Probe:* How often did you meet with [NAME OF COACH]?
   b. If yes:
      i. *Probe:* If you did meet with [NAME OF COACH], what were your meetings about?
      ii. *Probe:* If you did meet with [NAME OF COACH], did you find it helpful? Why or why not?
   c. If no:
      i. *Probe:* If you did not meet with [NAME OF COACH] weekly, why not?
7. Imagine U-PEACE is delivered online via Zoom. What would help to engage teens from your school in participating in online U-PEACE?
   a. *Probe:* What would motivate teens in your school to participate?
   b. *Probe*: What, if anything, would get in the way of teens participating in the online U-PEACE program?
   c. *Probe:* What, if anything, would get in the way of participating if the group was held after school?
   d. *Probe:* What, if anything, would get in the way of participating if the group was held during the summer?

8. Have we left anything out or not talked about something that you feel is important to cover in our interview today? Please explain.

FOR SAU:

1. What , if anything did you like about participating in this group?
2. What, if any, benefits did you receive from being in this group?
3. Any suggestions?
4. Imagine U-PEACE, the 2x/week treatment group to learn skills to manage strong emotions, is delivered online via Zoom. What would help to engage teens from your school in participating in online U-PEACE?
   a. *Probe:* What would motivate teens in your school to participate?
   b. *Probe*: What, if anything, would get in the way of teens participating in the online U-PEACE program?
   c. *Probe:* What, if anything, would get in the way of participating if the group was held after school?

   d. *Probe:* What, if anything, would get in the way of participating if the group was held during the summer?

5. Have we left anything out or not talked about something that you feel is important to cover in our interview today? Please explain.
   e.

Thank you very much once again for taking the time to talk to me about your thoughts and experience with the U-PEACE program. We will be using the information to inform the development and modification of the U-PEACE to increase its usefulness to schools and diverse adolescents and their caregivers.
