# Supplementary material for: An Iteratively Adapted Transdiagnostic Prevention Program for Diverse High School Settings (U-PEACE): Protocol for a Randomized Controlled Trial
Source: JMIR Res Protoc. 2025 Sep 24;14:e74080. doi: 10.2196/74080 (PMC12508673; doi:10.2196/74080)
Supplement: Multimedia Appendix 5 [file resprot_v14i1e74080_app5.pdf]

UNITED STATES DEPARTMENT OF EDUCATION
INSTITUTE OF EDUCATION SCIENCES

EDUCATION RESEARCH GRANTS

PEER REVIEW PANEL SUMMARY STATEMENT
(PRIVILEGED COMMUNICATION)

**Application Number:**	R305A210102
**Research Topic:**	Social and Behavioral Context for Academic Learning
**Project Type:**	Development and Innovation
**Meeting Dates:**	2/4/2021–2/5/2021
**Principal Investigator:**	Jill Ehrenreich-May
**Institution:**	University of Miami
**Project Title:**	Brief, Transdiagnostic Care to Support Academic Learning in Adolescents With Emotional Disorder Symptoms

Overall Score: 2.10
(1=outstanding; 5=poor)

Total Federal Funds Requested: $1,792,931.00
Duration of Support Requested: 3 Years

Evaluation Criteria Rating Scale: 1 (poor) to 7 (excellent)

The panel reviewed the application on the basis of the published evaluation criteria and rated it as described below.

Criteria Description	Score
Significance	5.1
Research Plan	5.1
Personnel	7.0
Resources	7.0
Dissemination	6.9

REVIEWER A:

Overall Description of this Application

The aim of this resubmitted Development and Innovation study is to develop and validate the Unified Protocol for Preventing Emotional and Academic Challenges in Education (UP-PEACE). UP-PEACE components will be based on the Unified Protocol for Transdiagnostic Treatment for Emotional Disorders in Adolescents (UP-A), which is a manualized nonschool-based empirically supported intervention used to treat youth depression and anxiety. Unlike UP-A, UP-PEACE will be designed specifically for use in school settings and is intended to serve as a Tier 3 intervention within existing MTSS/RTI systems. Intervention components include several well-known and empirically validated strategies (e.g., cognitive reappraisal, problem-solving, mindfulness, exposure, behavioral activation). The theory of action hypothesizes that UP-PEACE will alleviate internalizing symptoms and improve the emotion regulation of targeted students, and this in turn is hypothesized to impact academic outcomes through improvements in attendance and academic engagement. Investigators intend to first conduct qualitative evaluations intervention content with key stakeholder groups, followed by a small-scale pilot to determine feasibility, and ending with a pilot randomized control trial (RCT) to document intervention effectiveness.

Critique of this Application by Review Criteria for Scientific Merit

Significance: Does the applicant address the recommendations described in the Significance section for the project type under which the applicant is submitting the application?

Major Strengths: A strength of this study lies in the fact that primary components are based largely on the UP-A program that has been empirically demonstrated to be effective in addressing internalizing symptoms. Proposed components are well-known, empirically validated cognitive-behavioral and/or behavioral strategies. The literature review is well written and comprehensive. The applicants make a strong and compelling case for the need for programs such as the one proposed. The theory of change is clear, and evidence for implied causal linkages is strong. Examining the feasibility of intervention delivery by school health providers (e.g., school nurses) and exploration of remote intervention delivery options adds to the potential significance of the knowledge to be gained from the proposed study. The applicants were responsive to requests for additional elaboration regarding the potential benefits of UP-PEACE relative to existing school-based interventions for internalizing problems.

Major Weaknesses: Although investigating the feasibility of using school nurses for intervention delivery is a welcome addition, given the fact that this is a development study, the wisdom of relying on school nurses exclusively is questionable. The present application would be enhanced by treating the question of optimal/acceptable intervention delivery agents as an empirical question. Not including professionals most likely to deliver an intervention such as UP-PEACE (e.g., school psychologists, counselors, social workers) detracts from the potential significance of the proposed study.

Research Plan: Does the applicant address the recommendations described in the Research Plan section for the project type under which the applicant is submitting the application?

Major Strengths: The applicants have been highly responsive to concerns raised in prior reviews with regard to the research plan. Procedures for calculating reliable change indices have been clarified in the present version of the application, measures with questionable reliability have been removed, and the measurement protocol streamlined in order to address concerns regarding participant response fatigue. The applicants clarified questions raised in prior reviews regarding cost-effectiveness analysis and clearly stated they intend to conduct a cost analysis only. The applicants were highly responsive to concerns related to monitoring fidelity of implementation and field and have introduced additional protocols to accomplish this. Although the total number of assessments was reduced, the measurement plan remains strong and includes a thoughtful selection of well-validated measures and mixture of assessment methods. Procedures related to recruitment, consent, and the proposed timeline of study activities are clear and realistic. Data analytic procedures are clear, sophisticated, and appropriate for addressing study research questions. The applicants have been highly responsive to concerns related to meeting study recruitment quotas and plan to conduct year round recruitment for the final RCT in light of concerns raised in prior reviews.

Major Weaknesses: The applicants have removed proposed tests of mediation from the present application due to concerns regarding power. It would have arguably been preferable to propose some manner of evaluation, even if underpowered. The absence of preliminary evidence for the theory of action reduces the potential value of the proposed study.

Personnel: Does the applicant address the recommendations described in the Personnel section for the project type under which the applicant is submitting the application? Do the principal investigator, project director, and other key personnel possess appropriate training and experience and will they commit sufficient time to competently implement the proposed research?

Major Strengths: The research team comprises experienced investigators who are highly qualified to conduct the work proposed. Coinvestigators provide complementary expertise related to the intervention under consideration. The roles and responsibilities of team members are clear. The team includes investigators with substantial methodological expertise and experience in conducting the type of complex analyses proposed. Concerns raised in previous reviews with regard to limited study team expertise in conducting qualitative analyses have been adequately addressed in this revision. Principal Investigator (PI) time commitment (25 percent AY; 50 percent SU) is adequate to oversee proposed study activities.

Major Weaknesses: No major weaknesses were noted.

Resources: Does the applicant address the recommendations described in the Resources section for the project type under which the applicant is submitting the application? Does the applicant have the facilities, equipment, supplies, and other resources required to support the proposed activities? Do the commitments of each partner show support for the implementation and success of the project?

Major Strengths: The University of Miami is a well-resourced R1 institution well positioned to conduct the proposed research activities. Letters of support, although not required for this cycle, clearly document on-the-ground support for the project.

Major Weaknesses: No major weaknesses were noted.

Dissemination: Does the application address the recommendations described in Appendix A: Dissemination History and Plan? Does the applicant present a dissemination plan that is tailored to the purpose of the project type and designed to reach a wide range of audiences? Does the applicant describe a dissemination history that demonstrates past success in sharing results of education research widely and appropriately?

Major Strengths: The dissemination plan is appropriate given the nature of the proposed project and is designed to reach a wide range of stakeholder audiences with a vested interest in study outcomes. Collectively, the research team has demonstrated success in sharing the results of their work using a variety of modalities.

Major Weaknesses: No major weaknesses were noted.

Summary of Application Critique

This resubmitted Development and Innovation application is responsive to many concerns raised in prior reviews. The proposed intervention addresses the needs of students with internalizing disorder which is significant given there are few feasible and effective school-based Tier 3 options for this population. The proposed intervention builds on the success of UP-A, and is based on a compelling and well-supported theory of action. The applicant’s decision to focus exclusively on school nurses as an intervention delivery agent, while innovative and worthy of investigation, limits the significance of the proposed study as it substantially deviates from common school practices and procedures related to the delivery of interventions such as UP-PEACE. School psychologists, counselors, and/or social workers are most likely to deliver UP-PEACE in real-world settings, and the new focus on school nurses is a potentially problematic feature of this revised application. Similarly, the decision to completely drop formal tests of mediation of causal pathways implied by the intervention theory of action is problematic given this is an important goal of development-focused research. Nonetheless, the research team comprises highly qualified researchers with adequate expertise and access to resources necessary to accomplish study objectives. The dissemination plan is comprehensive and likely to be successful in reaching a diverse array of stakeholder audiences.

REVIEWER B:

Overall Description of this Application

This application consists of a 3-year Development and Innovation project focused on developing and conducting a pilot test of UP-PEACE, an indicated school-based program designed to support students' mental health. The work targets students aged 13 to 18 from underserved, low income, and diverse communities. The project involves modifying an existing program, examining its feasibility, and conducting an RCT as a pilot.

Critique of this Application by Review Criteria for Scientific Merit

Significance: Does the applicant address the recommendations described in the Significance section for the project type under which the applicant is submitting the application?

Major Strengths: The applicants address a serious and growing concern: The mental health of students, with special attention given to emotional concerns of depression and anxiety. They build upon their own work in this area by improving upon existing models to make them more suitable for schools and racially and socioeconomically diverse populations. They incorporate strong theory and evidence from best practices, mental illness diagnosis, and treatment. The applicants' theory of change is clearly articulated. The study is also timely given the stress that the pandemic and social unrest is having on students and their families.

Major Weaknesses: The applicants discuss the potential value of using nurses to implement mental programs like UP-PEACE and describe literature that suggests this to be the case. However, it seems that school social workers or psychologists would be much better suited for such a role. In their response to prior reviewers, the applicants note this decision, but they provide little rationale.

Research Plan: Does the applicant address the recommendations described in the Research Plan section for the project type under which the applicant is submitting the application?

Major Strengths: The applicants designed a strong study that uses mixed methods to develop and provide initial evidence of effectiveness. One important strength is their recruitment of schools and professionals serving a sample that comes from low income and racially diverse backgrounds. The applicants adequately address prior review concerns such as providing more details about multilevel models, missing data, reliability of measures, test fatigue, and other concerns.

Major Weaknesses: The results from their power analysis are confusing. For example, they state that "the study will have power of B=.33 to detect a medium effect (d=.50)...." But power is 1-B, so are they reporting B or power? If what they are reporting is power, assuming B = power, the applicants report that a fully powered study can detect an effect size of .94. This is a very large effect, and they state that this is smaller than previous tests of UP-A. However, they do not mention what those values were.

Personnel: Does the applicant address the recommendations described in the Personnel section for the project type under which the applicant is submitting the application? Do the principal investigator, project director, and other key personnel possess appropriate training and experience and will they commit sufficient time to competently implement the proposed research?

Major Strengths: The research team has deep experience in the content of mental health and schools. The PI has prior experience managing federal grants as a PI for three NIH grants. All team members have adequate time dedicated to the project.

Major Weaknesses: No major weaknesses were noted.

Resources: Does the applicant address the recommendations described in the Resources section for the project type under which the applicant is submitting the application? Does the applicant have the facilities, equipment, supplies, and other resources required to support the proposed activities? Do the commitments of each partner show support for the implementation and success of the project?

Major Strengths: The University of Miami and the John T. MacDonald Foundation School Health Initiative provide adequate resources for carrying out the work.

Major Weaknesses: No major weaknesses were noted.

Dissemination: Does the application address the recommendations described in Appendix A: Dissemination History and Plan? Does the applicant present a dissemination plan that is tailored to the purpose of the project type and designed to reach a wide range of audiences? Does the applicant describe a dissemination history that demonstrates past success in sharing results of education research widely and appropriately?

Major Strengths: The research team has a demonstrated history of disseminating information regarding the UP-A for the general public and the mental health community. Currently, a website is maintained for disseminating information regarding all research related to UP-A and UP manuals for children (UP-C). The dissemination plan considers dissemination to various audiences in accessible language.

Major Weaknesses: No major weaknesses were noted.

Summary of Application Critique

Overall, this is a strong study that addresses a serious and growing problem. The study is especially timely now. The applicants were responsive to reviewer critiques and have improved many aspects of study design. The main limitations of the study concern who the appropriate implementers of UP-C are and the lack of detail regarding the power analyses.

SUMMARY OF PANEL DISCUSSION:

At the panel session, the primary reviewers presented their individual critiques of the application. The panel listened to the primary reviewers' discussion of the strengths and weaknesses of the research plan and to their evaluation of the significance of the proposed project, the qualifications of the research team, the adequacy of the resources, and the quality and appropriateness of the dissemination history and plan. After hearing the primary reviewers' perspectives, the panel discussed the following aspects of the application.

Panelists discussed the choice of nurses, particularly given the scarcity of them in many schools. More information about the feasibility of using school nurses would strengthen the application, whether they would be available during the summer, and whether they are the best population to deliver the intervention (e.g., will it fall within their scope of training and practice). A panelist asked for more information about how the program might or might not work similarly across ethnic populations and what steps will be taken to recruit a diverse sample. A panelist noted the move to telehealth is a strength, but asked for clarification about what changes might need to be made for the move to telehealth.

(1) Title: Brief, Transdiagnostic Care to Support Academic Learning in Adolescents with Emotional Disorder Symptoms
Topic and Project Type: 84.305A; Education Research: Social and Behavioral Context for Academic Learning; Development and Innovation Project

(2) Project Summary

Purpose: The purpose of this mixed-methods evaluation project is to develop, refine, and pilot test a school-based, indicated prevention program to address emotion regulation challenges faced by racially- and culturally-diverse adolescents with anxiety and depressive symptoms living in lower-income communities, the Unified Protocol for Preventing Emotional and Academic Challenges in Education (UP-PEACE).
Project Activities: The proposed project involves developing and testing the feasibility of a modified version of the Unified Protocol for Transdiagnostic Treatment of Emotional Disorders in Adolescents (UP-A), an evidence-based intervention for anxiety and depression in adolescents via telehealth. The intervention will be an indicated prevention program targeting emotion regulation, which we hypothesize will result in the reduction of clinical symptoms and improved academic achievement. The goal of this mixed methods evaluation project is to iteratively collect and integrate qualitative and quantitative data to develop UP-PEACE as a program that can be easily implemented, is scalable and sustainable in high schools and with racially- and culturally-diverse youth being served in school-located healthcare settings. The proposed project consists of three phases (Pre-Case Series Focus Groups; Case Series; Randomized Controlled Trial). Adolescent participants will be recruited from three public high schools in Miami-Dade County affiliated with the University of Miami's John T. MacDonald School Health Initiative. A total of 181 participants (92 adults and 89 adolescents) will be recruited during the course of the proposed study. Qualitative, quantitative and cost outcomes will be used to inform a fully-powered, randomized, effectiveness trial of the UP-PEACE program in school health settings.
Products: The proposed study will result in a fully developed version of the UP-PEACE indicated prevention program for anxiety and depression in racially- and culturally-diverse adolescents, including its clinician manual and participant workbook, clinician training and fidelity assessment materials suitable for use in both English and Spanish during future trials.

(3) Structured Abstract

Setting: This study will take place in high schools in Miami-Dade County, Florida.
Population/Sample: The participating sample includes a diverse sample of students (ages 13-18) and school personnel, along with parents of students with higher or "indicated" risk for anxiety and depressive symptoms, and clinicians. Following a series of focus groups with key stakeholders that will give qualitative feedback on the development of UP-PEACE, an iterative case series with 12 adolescents will be conducted. Approximately 72 adolescents in three schools will then participate in the pilot randomized control trial (RCT). Pending feedback from key stakeholders, we anticipate that UP-PEACE will be provided by school nurses working in school-located, integrated pediatric primary care settings.
Intervention/Assessment/Factors: The UP-PEACE program will be modified for school settings from the existent Unified Protocol for Transdiagnostic Treatment for Emotional Disorders in Adolescents (UP-A), a transdiagnostic form of cognitive behavior therapy, using qualitative feedback from key stakeholders. The UP-A presents evidence-based practices for emotional disorders in a transdiagnostic context**:** cognitive strategies (e.g., reappraisal, problem-solving),

mindfulness-based strategies (e.g., non-judgmental awareness), behavioral strategies (e.g., exposure, behavioral activation, opposite action). Potential modifications include an abbreviated, rolling group structure, telehealth delivery, in-school behavioral skill practice, and streamlined materials for school clinicians. In planned modifications to the UP-A, we plan to also consider the context in which UP-PEACE is provided (school-located, integrated pediatric primary care), school mental health provider type delivering the program (school nurses) and the lower-income, highly diverse communities from which the sample of adolescents for this study will be drawn.

Control Condition: Services as usual (SAU) in the school-located, integrated pediatric primary care clinics in which the study will take place will serve as the comparison condition for the RCT.

Research Design and Methods: Our mixed methods evaluation design involves multiple iterative phases, with the integration of qualitative and quantitative data occurring within and across phases. As a first step, we will develop UP-PEACE as an acceptable indicated prevention program for use in schools with the input of focus groups with distinct stakeholders (school mental health providers [including school nurses], school administrators, adolescents, teachers and parents). A case series consisting of two iterations will then be conducted with adolescents exhibiting elevated anxiety and/or depression symptoms, to evaluate preliminary feasibility and targets for further modification. Following each case series, the research team will conduct qualitative interviews and focus groups to obtain feedback on any factors influencing the implementation of the UP-PEACE within schools. The combination of quantitative and qualitative data collected concurrently during this phase will allow us to better understand how individual treatment response may be related to perceived participation barriers and facilitators. Subsequently, we plan to conduct a pilot randomized controlled trial (RCT) in which adolescents, who will be randomly assigned to either UP-PEACE or services as usual in the in the school-located, integrated pediatric primary care clinic at their school (SAU). Following the RCT, we will conduct another round of qualitative data collection from purposefully sampled adolescents and parents as well as from school stakeholders who will provide their input on how to enhance the intervention, its implementation potential and scalability. Qualitative and quantitative data from all stages of the investigation, plus preliminary cost effectiveness data, will be used to prepare final procedures for a future, large-scale effectiveness trial.

Key Measures: Primary anxiety and depression symptom outcomes will be examined dimensionally using brief, easily administered outcome measures, including the Patient Health Questionnaire-9 and Generalized Anxiety Disorder 7-item Scale. Changes in the target mechanism, emotion regulation, along with academic achievement and academic problems will also be assessed.

Data Analytic Strategy: Qualitative thematic analysis will be used to identify themes and subthemes in focus group and interview data. Quantitative analyses will use multilevel modeling (MLM), to account for nesting of repeated measures within clients and treatment groups; given the small number of schools and therapists, these variables will be included as control variables and probed for a future, fully-powered trial. Mixed methods analyses will involve developing joint displays following the case series and RCT to merge qualitative and quantitative data (e.g., compare illustrative quotes for adolescents who improve vs. worsen/remain unchanged following the intervention).

Cost Analysis: A preliminary estimate of costs associated with the intervention and its implementation will be calculated as a tool for communication about UP-PEACE with stakeholders and to help prepare for future effectiveness research.
